# A small molecule inhibitor of HER3: a proof-of-concept study

**DOI:** 10.1042/BCJ20200496

**Published:** 2020-09-10

**Authors:** Audrey Colomba, Martina Fitzek, Roger George, Gregory Weitsman, Selene Roberts, Laura Zanetti-Domingues, Michael Hirsch, Daniel J. Rolfe, Shahid Mehmood, Andrew Madin, Jeroen Claus, Svend Kjaer, Ambrosius P. Snijders, Tony Ng, Marisa Martin-Fernandez, David M. Smith, Peter J. Parker

**Affiliations:** 1 Protein Phosphorylation Laboratory, The Francis Crick Institute, London, U.K.; 2 Hit Discovery, Discovery Sciences, R&D, AstraZeneca, Alderley Park, Macclesfield, U.K.; 3 Structural Biology Science Technology Platform, The Francis Crick Institute, London, U.K.; 4 Richard Dimbleby Department of Cancer Research, School of Cancer and Pharmaceutical Sciences, King's College London, Guy's Campus, London, U.K.; 5 Central Laser Facility, Research Complex at Harwell, Science and Technology Facilities Council, Rutherford Appleton Laboratory, Didcot, U.K.; 6 Protein Analysis and Proteomics Science Technology Platform, The Francis Crick Institute, London, U.K.; 7 Hit Discovery, Discovery Sciences, R&D, AstraZeneca, Cambridge, U.K.; 8 Emerging Innovations Unit, Discovery Sciences, R&D, AstraZeneca, Cambridge, U.K.; 9 CRUK KHP Centre, School of Cancer and Pharmaceutical Sciences, King's College London, Guy's Campus, London, U.K.

**Keywords:** allostery, drug screening, HER3, inhibitor, pseudokinases

## Abstract

Despite being catalytically defective, pseudokinases are typically essential players of cellular signalling, acting as allosteric regulators of their active counterparts. Deregulation of a growing number of pseudokinases has been linked to human diseases, making pseudokinases therapeutic targets of interest. Pseudokinases can be dynamic, adopting specific conformations critical for their allosteric function. Interfering with their allosteric role, with small molecules that would lock pseudokinases in a conformation preventing their productive partner interactions, is an attractive therapeutic strategy to explore. As a well-known allosteric activator of epidermal growth factor receptor family members, and playing a major part in cancer progression, the pseudokinase HER3 is a relevant context in which to address the potential of pseudokinases as drug targets for the development of allosteric inhibitors. In this proof-of-concept study, we developed a multiplex, medium-throughput thermal shift assay screening strategy to assess over 100 000 compounds and identify selective small molecule inhibitors that would trap HER3 in a conformation which is unfavourable for the formation of an active HER2–HER3 heterodimer. As a proof-of-concept compound, AC3573 bound with some specificity to HER3 and abrogated HER2–HER3 complex formation and downstream signalling in cells. Our study highlights the opportunity to identify new molecular mechanisms of action interfering with the biological function of pseudokinases.

## Introduction

Pseudokinases belong to the protein kinase superfamily, but are catalytically compromised. A recent comprehensive analysis of pseudokinase sequences across species, has revealed that they are present across a wide variety of organisms, from fungi and bacteria, to plants and animals, highlighting their fundamental role in physiology [1]. In vertebrates, 10% of kinases are pseudokinases, which account for around 60 proteins encoded by the human genome [2]. Despite displaying defective catalytic kinase activity, pseudokinases actively regulate various signalling pathways, playing an important role in many cellular processes.

Pseudokinases bear changes in key amino acids essential for catalytic kinase activity, impairing their ability to bind to either divalent metal cations or nucleotides, or to catalyse transfer of the phosphate group onto substrates [3,4]. However, 50% of these inactive kinases can still bind to ATP, and this appears to be a prerequisite for their function [5,6]. For example, ATP-bound STRAD*α* adopts an ‘active’ closed conformation required for its oligomerisation with MO25 and LKB1 to promote tumour-suppressor activity of LKB1 [7,8].

Through their function as allosteric modulators of other enzymes’ activities, pseudokinases play an essential role in regulating cell signalling [9]. Thus, in the JAK tyrosine kinase family, the catalytic activity of the kinase domain JH1 is negatively regulated by the intramolecular pseudokinase domain JH2 [10]. The kinase suppressors of Ras 1 and 2, KSR1/2, are pseudokinases which act as allosteric regulators of RAF kinase activity and as scaffold anchors of the signalling hub Raf-MEK-ERK [11–13]. Some pseudokinases function as spatial modulators, controlling sub-cellular localisation of substrates [3,5], while others regulate protein trafficking and degradation, like the Tribble family which is involved in COP1-dependent ubiquitylation [14].

Since pseudokinases have a diverse range of physiological roles, disruption of their function is associated with a wide variety of human pathologies, including metabolic and neurological disorders, autoimmune diseases, cardiomyopathies and cancers [2,9]. They thus represent attractive drug targets for therapeutic intervention and a growing number of them have been explored for the development of small molecule or biological agents [9,15]. Among pseudokinases, HER3 has emerged as a potential therapeutic target in cancer.

HER3 is a member of the epidermal growth factor receptor (EGFR) family, which comprises four closely related tyrosine kinase receptors: HER1 (EGFR), HER2, HER3 and HER4. Ligand binding to these receptors initiates conformational rearrangements, which allow asymmetric kinase domain homo or heterodimers to form between two EGFR family members wherein, one kinase domain allosterically activates the other [16,17]. HER2 and HER3 are non-autonomous receptors and physiologically only form signalling competent heterodimers, as HER2 lacks the capacity to interact with ligand, whereas HER3, with its defective kinase domain, only retains an allosteric function. HER3 is an essential allosteric activator of EGFR members, in particular of HER2 which is its preferred heterodimerisation partner [18].

Under physiological conditions, EGFR family members are potent mediators of cell growth and have an important role in embryonic development and tissue homeostasis. But, their deregulation is associated with the development and progression of many types of cancer [18,19]. Thus, HER3 deregulation plays a crucial role in many oncogenic processes [20]. HER3 itself is overexpressed and associated with poor prognosis in ovarian and breast cancers [21]. In HER2-dependent breast cancer, HER3 has been shown to be essential for HER2 transforming properties and tumour cell survival [18,22]. Moreover, several gain of function somatic mutations in HER3 extracellular domain (promoting ligand-independent activation) and intracellular domain (enhancing its allosteric ability) have been described to support tumorigenesis in various types of cancers, including colon and gastric cancers [23,24]. HER3 signalling up-regulation has also been shown to promote resistance to EGFR and HER2-targeted therapies [25–27].

Given its importance in activating oncogenic signalling pathways and in acquired resistance to targeted therapies, HER3 represents an attractive target in cancer and several therapeutic strategies against this pseudokinase have been reported [9]. They are mostly antibody-based, blocking ligand binding to HER3, or preventing its dimerisation with other EGFR receptors or triggering its internalisation [20,28,29]. A very few pharmacological approaches to targeting HER3 have been developed, essentially interfering with its expression [30], including modified ATP-competitive molecules binding to HER3 to induce its proteasomal degradation [31].

For many pseudokinases, like HER3, conformational rearrangements are a prerequisite to their allosteric function. Exploiting this, to develop compounds which would lock pseudokinases in a non-functional conformation and prevent their interaction with their binding partners, might be an effective therapeutic strategy [9]. Thus, the allosteric function of KSR2 can be modulated by an ATP-competitive inhibitor which stabilises a conformation of KSR2 that is incompatible with its heterodimerisation with BRAF [32]. However, as has been observed for their active counterparts, such as PKC*ε* [33], PKB/Akt [34], BRAF [35–38] and as we recently showed for HER2 [39], targeting the nucleotide-binding pocket of pseudokinases, may result in enhancing their allosteric function rather than inhibiting it [40]. We indeed recently reported that for HER3, occupancy of its nucleotide-binding pocket is essential for its allosteric function and that, the ATP-competitive inhibitor, bosutinib stabilises HER3 and promotes its dimerisation with HER2 [39]. Bosutinib was one of a series of HER3 ligands also identified in a competitive screen for the selectivity of kinase binding amongst known kinase inhibitors [41].

Hence, there is scope for the development of inhibitors with alternative mechanisms of action that would disrupt the functional conformation of pseudokinases and interfere with their allosteric function preventing signalling-capable protein–protein interactions. To validate the concept of allosteric inhibitors of pseudokinases, we report here a differential scanning fluorimetry (DSF) screening strategy to identify small molecules capable of blocking HER3 in a conformation that prevents the formation of an active signalling heterodimer with HER2. Here, we describe a study yielding a proof-of-concept compound that binds HER3 to inhibit HER3 allosteric activator function.

## Materials and methods

### Reagents and antibodies

Lapatinib was purchased from Selleckchem, bosutinib from LC Labs, MG-132 from Sigma, BX-912 from Cambridge Bioscience, LY294002 and BIM1 from Merck Chemicals, staurosporine from ThermoFisher Scientific and recombinant human heregulin *β *− 1 (NRG) (cat.no.100-03) from Peprotech. BLU577 and CRT0066854 were kindly provided by Dr. Jon Roffey Cancer Research Technology, U.K. Anti-phospho-HER3 (Y1289) (cat.no.4791), anti-HER3 (cat.no.12708), anti-phospho-HER2 (Y1248) (cat.no.2247), anti-phospho-HER2 (Y877) (cat.no.2241), anti-phospho-HER2 (Y1221/1222) (cat.no.2249), anti-phospho-Akt (Ser473) (cat.no.4060), anti-panAkt (cat.no.2920), anti-phospho-ERK1/2 (T202/Y204) (cat.no.4370), anti-ERK1/2 (cat.no.4696) were purchased from Cell Signaling Technology (CST). Anti- HER2 for Western blot (cat.no.ab16901) and anti-HER2 for FRET (e2-4001+3B5) were purchased from AbCam and ThermoFisher Scientific, respectively. Anti-*α*-tubulin (cat.no.T5168) was from Sigma. IRDye secondary antibodies and Odyssey blocking buffer were from Li-Cor Biosciences.

For FLIM-FRET experiments, anti-HER2 and anti-HER3 primary antibodies were directly labelled at particular dye/protein (D/P) ratio for optimal performance of the assay (donor 1 : 1 and acceptor 1 : 3) with Cy5 (Amersham GE) and Alexa546 (X546, ThermoFisher Scientific) fluorophores, respectively, according to the manufacturers’ protocol.

For tracking of HER2–HER3 complexes and HER2–HER3 clustering experiments, NRG was conjugated with CF640R SE (Biotium) or Alexa647 (ThermoFisher Scientific) according to manufacturer's instructions. HER2 and HER3 Affibody ligands were used to label the non-activated states of the receptors, HER2 was from Affibody Inc. and the plasmid encoding HER3 Affibody was a gift from John Löfblom. The conjugation of Alexa488 (ThermoFisher Scientific), Alexa647 or CF640R maleimide dyes to HER2 and HER3 Affibody ligands was performed according to the manufacturer's instructions to achieve a ∼1 : 1 ratio of dye : ligand.

### Chemical screening library

A library of 107 008 diverse small molecule compounds (AstraZeneca) in DMSO was arrayed, in pools of four compounds, in MicroAmp Optical 384-well reaction plates (Applied Biosystems, ThermoFisher Scientific). The compounds were selected as a representative subset of the entire corporate 2.2 million compound screening library to provide maximal chemical diversity, while retaining good physicochemical properties (see https://openinnovation.astrazeneca.com/target-innovation.html#).

### Cell culture

All cell lines were sourced from the Francis Crick Institute's Cell services facility, where they were tested negative for mycoplasma and authenticated by STR profiling. Human breast cancer SK-BR-3 cells were grown in Dulbecco's modified Eagle's medium (DMEM, Gibco ThermoFisher Scientific) and Chinese Hamster Ovary (CHO) cells in nutrient mixture F12 Ham with l-glutamine and sodium bicarbonate (Sigma). All media were supplemented with 10% (v/v) fetal bovine serum (FBS) (Gibco ThermoFisher Scientific), penicillin and streptomycin (Gibco ThermoFisher Scientific). All cells were maintained at 37°C and in 10% CO_2_.

### Plasmid transfection

CHO cells were plated at 0.85 × 10^5 ^cells/well in 12-well plates, grown for 24 h before transfection with the indicated plasmids using Viafect (Promega), according to the manufacturer's protocol.

### Site-directed mutagenesis

HER2 full length wild-type HA tagged pcDNA3.1 plasmid was a gift from Prof. Yosef Yarden (The Weizmann Institute). Site-directed mutagenesis was completed using the following primers, Fwd 5′atcatctctgcggtggttgAcattctgctggtcgtggtc and Rev 5′ gaccacgaccagcagaatgTcaaccaccgcagagatgat, and Fwd 5′gtggttggcattctgctggAAgtggtcttgggggtggtc and Rev 5′gaccacccccaagaccacTTccagcagaatgccaaccac, to generate G660D and V664E mutants, respectively. PCR reactions were performed as follows, 95°C 30 s, 18 cycles of 95°C 30 s, 50°C 1 min and 68°C 20 min, using Pfu Turbo polymerase (Agilent), according to the manufacturer's recommendations. All clones were sequence verified using the following primers HER2 Fwd 5′aagtttccagatgaggagg, CMV Fwd 5′cgcaaatgggcggtaggcgtg and BGH Fdw 5′tagaggcacagtcgagg.

### Recombinant protein expression and purification

HER3 kinase domain (residues G684-D1020 [42]) and HER2 kinase domain (S703-G1029, M706A, Q711L, M712L) constructs were kindly provided by Prof. Mark Lemmon, University of Pennsylvania and Dr. Caroline Truman, AstraZeneca, respectively. Constructs were cloned into the pFastBac vector and used to generate baculovirus using standard published protocols (ThermoFisher Scientific). Routinely, 500 ml suspension cultures of Sf21 cells at a density of 1.5 × 10^6 ^cells/ml were infected with virus encoding the HER proteins and allowed to grow for a further 3 days at 27°C and 110 rpm. Cells were then harvested by centrifugation and stored at −80°C.

Cell pellets were resuspended in 20 ml lysis buffer containing 50 mM HEPES pH 8.0, 300 mM NaCl, 1(v/v)% Triton X-100, 10(v/v)% glycerol, 10 mM *β*-glycerophosphate, 10 mM benzamidine, 0.5 mM EDTA, 1 mM NaF, 1 mM DTT and protease inhibitors. The suspension was then sonicated to ensure complete lysis. Insoluble material was removed by centrifugation at 30 000 rpm and the soluble material incubated with 200 µl bead volume of washed and equilibrated NiNTA resin (Qiagen) for 1 h at 4°C with constant rolling. The resin was then washed extensively with buffer containing 50 mM HEPES pH 8.0, 300 mM NaCl, 5% glycerol, 25 mM imidazole, 1 mM DTT, 5 mM MgCl2 and 200U DNAse. HER proteins were eluted using the same buffer supplemented with 200 mM imidazole as 3 × 1 ml washes. Eluted protein was then concentrated to 0.5 ml using a Vivaspin protein concentrator with a molecular mass cut-off of 10 000 kDa. The concentrated proteins were then applied to a S200 10/300 size exclusion column (GE Healthcare) equilibrated with wash buffer. Peaks were analysed by SDS–PAGE and fractions containing HER proteins were combined, aliquoted as required, snap frozen in liquid nitrogen and stored at −80°C.

### Differential scanning fluorimetry screening

Thermal shift assays were performed using a QuantStudio7 Flex Real-Time PCR instrument (Applied Biosystems, ThermoFischer Scientific) scanning from 15°C to 75°C in 0.5°C increments every 30 s after an initial incubation at 15°C for 1 min. Fluorescence was measured at the end of each 30 s period with an excitation wavelength of 470 nm and an emission wavelength of 620 nm. HER3 and HER2 kinase domain recombinant proteins were diluted in 50 mM Hepes pH 7.5, 150 mM NaCl, 5% glycerol and 1 mM DTT to a concentration of 1.5 μM and then incubated with the indicated concentration of compounds in a total reaction volume of 10 μl, with a final concentration of 2.5X SYPRO Orange (ThermoFisher Scientific) as fluorescence probe. Each assay plate included neutral (1.2% or 0.3% DMSO) and positive (200 μM ATP/10 mM MgCl2 for HER3, 1 μM lapatinib for HER2) control wells.

### Data analysis of the differential scanning fluorimetry screen

The data were analysed using the Genedata Screener® software, normalised to a neutral control. The cut-off for activity was based on 3×SD of neutral control in each assay. Compounds that showed >3°C shift of the *T*_m_ for HER3 and >2°C shift of the *T*_m_ for HER2, as compared with the DMSO control were selected as binding hits. Rz factor was calculated from Rz = 1 − (3×rSD HER3 + ATP + 3×rSD ApoHER3)/(median HER3 + ATP-median ApoHER3).

### HDX-MS

HER3 protein was prepared at 9 µM in purification buffer (50 mM Hepes pH 7.5, 150 mM NaCl, 5% glycerol and 1 mM DTT). A volume of 3 μl of protein was diluted to 7 μl in purification buffer. Deuterated samples were prepared by 19-fold dilution into the deuteration buffer containing D2O. After incubation for 10 s, 1 min and 6 min at 25 °C, samples were mixed with equal volumes of cold quench-buffer of 30 mM Tris(2-carboxyethyl)phosphine (TCEP), 50 mM HCl followed by flash freezing in liquid nitrogen. Prior to analysis, the samples were thawed quickly and manually loaded. For protein–ligand complex samples, protein samples were mixed with AMP-PNP, bosutinib or AC3573 compound at a final concentration of 100 μM. Hydrogen-deuterium exchange mass spectrometry (HDX-MS) was performed using a Waters HDX platform composed of a nano-Acquity ultra-performance liquid chromatography coupled to a Synapt G2-Si (Waters) mass spectrometer as previously described [43]. Leucine enkephalin at a continuous flow rate of 5 µl/min was sprayed as a lock mass for mass correction. Proteins were digested in-line using a pepsin immobilised column at 20 °C. The peptides were trapped on a peptide trap at a flow rate of 150 µl/min for 3 min and further separated using a reverse-phase C18 column with a linear gradient 5–80% of water and acetonitrile, both supplemented with 0.1% formic acid for 7 min at a flow rate of 40 µl/min at 0 °C. Peptide sequence coverage and deuterium uptake data was analysed by using PLGS and DynamX programs (Waters), respectively.

### FLIM-FRET

A total of 0.3 × 10^5^ SK-BR-3 cells were seeded on glass coverslips in 24-well plates in complete medium. The medium was changed 3 days later to FBS free medium and cells were serum-starved for 24 h. Inhibitors (lapatinib, 1 μM, and AC3573, 30 μM) were added 1 h before treatment with NRG at 10 nM for 15 min. Cells were then fixed with 4% paraformaldehyde for 15 min, washed with TBS and stained with anti-HER3-X546 alone (donor only) or with anti-HER3-X546 and anti-HER2-Cy5 antibodies (donor plus acceptor). Samples were imaged on a customised ‘open’ microscope fitted with an automated FLIM system [44]. Time-domain fluorescence lifetime images were acquired via time-correlated single photon counting (TCSPC) at a resolution of 256 by 256 pixels, with 256 time bins and 100 frames accumulated over 300 s; conventional wide-field fluorescence images were acquired at a resolution of 1024 by 1024 pixels on a CCD camera (Hamamatsu 1394 ORCA-ERA) with an exposure time of 200 ms, as previously described [45]. For each treatment group a ‘donor’ image and a ‘donor with acceptor’ image were acquired. FLIM analysis was performed with the TRI2 software (Version 2.7.8.9, Gray Institute, Oxford) as described previously [46,47]. The FRET efficiency for each cell was calculated according to the equation FRET eff = 1 − (*τ*DA/*τ*D), where *τ*D is the average lifetime of Alexa546 in the absence of Cy5 from the donor image and *τ*DA is the average lifetime of Alexa546 in the presence of Cy5, from the ‘donor with acceptor’ image.

### Tracking of HER2–HER3 complexes

CHO cells were plated at 1.8 × 10^5 ^cells/dish on 1% BSA-coated 35 mm glass-bottom dishes (Matek) and grown for 24 h before transfection with HA-HER2-WT and HA-HER3-WT full-length plasmids using Viafect (Promega), according to the manufacturer's protocol. Forty-eight hours post-transfection, cells were serum-starved for 1 h, then treated for 1 h with 30 μM AC3573 compound or 0.3% DMSO in serum-free medium. Cells were then labelled with 0.5 nM HER2-Alexa488 Affibody and 15 nM HER3-CF640R Affibody or 14 nM NRG-CF640R for 7 min at 37°C and washed with serum-free medium before prompt imaging. Single-molecule images were acquired using an Axiovert 200M microscope with TIRF illuminator (Zeiss, U.K.), with a 100× oil-immersion objective (*α*-Plan-Fluar, NA = 1.45; Zeiss, U.K.) and an EMCCD (iXon X3; Andor, U.K.). The 488 nm and 642 nm lines of a LightHub laser combiner (Omicron Laserage GmbH) were used to illuminate the sample and an Optosplit Image Splitter (Cairn Research) was used to separate the image into its spectral components as described previously [48]. The field of view of each channel for single-molecule imaging was 80 × 30 µm. All single-molecule time series data were analysed using the multidimensional analysis software described previously [49]. Calculation of colocalisation and τON were performed as previously described [50].

### HER2–HER3 clustering assay

SK-BR-3 cells were seeded at 2 × 10^5 ^cells/dish on 1% BSA-coated 35 mm glass-bottom dishes (Matek). Four days later, cells were serum-starved in phenol red-free medium for 2 h, then treated for 1 h with 30 μM AC3573 compound, 1 μM Lapatinib or 0.1% DMSO. Cells were washed with cold PBS and labelled in cold PBS 1% BSA with 150 nM HER2-Alexa488 Affibody and 10 nM NRG-Alexa647 or 50 nM HER3-Alexa647 for 1 h at 4°C with or without drugs as indicated. Cells were washed with cold PBS and fixed with 3%PFA + 0.5% glutaraldehyde in cold PBS. Single fluorophore localisations in equatorial regions of cells were imaged in TIRF mode using a Zeiss Elyra super-resolution microscope with a 100× oil-immersion objective; 488 nm and 640 nm lasers were used to excite Alexa488 and Alexa647 fluorophores bound to HER2 and HER3 receptors, respectively, and, as required, a 405 nm laser was used to induce additional fluorophore blinking. For the full data acquisition protocol see [51].

### Compound kinase profiling

*In vitro* profiling of AC3573 at 1 μM was conducted by ThermoFisher Scientific. http://assets.thermofisher.com/TFS-Assets/BID/Methods-&-Protocols/20180123_SSBK_Customer_Protocol_and_Assay_Conditions.pdf

### Immunoblotting

SK-BR-3 cells were plated at 1.7 × 10^5 ^cells/well in 24-well plates and grown for 24 h in complete medium before serum-starvation for 16 h. Post-transfection (24 h) CHO cells were serum-starved for 16 h. Cells were then incubated with small molecule compounds or commercial inhibitors, at the indicated concentrations, for 1 h in serum-free medium. SK-BR-3 cells were then stimulated or not, as indicated, with 10 nM NRG for 15 min in serum-free medium.

Whole-cell lysates were generated using 50 mM Tris–base pH8, 150 mM NaCl, 5 mM EGTA, 1% (v/v) Nonidet-P40 supplemented with protease (complete mini-tablets, Roche) and phosphatase (Calbiochem) inhibitors, briefly sonicated and cleared by centrifugation at 13 000 rpm for 15 min at 4°C. Samples were boiled for 10 min in NuPAGE LDS Sample Buffer (ThermoFisher Scientific) with 100 mM DTT. Subsequently, total proteins were resolved by SDS–PAGE on 3–8% gradient gels (ThermoFisher Scientific) followed by transfer onto low fluorescence PVDF membrane (Millipore). After blocking in Odyssey blocking buffer, primary antibodies were incubated in Odyssey blocking buffer with 0.02% (v/v) Tween-20 and proteins were detected using IRDye secondary antibodies and scanned on an Odyssey CLx imager (Li-Cor). Immunoblots were quantified using ImageStudioLite software (Li-Cor Biosciences).

### Statistical analysis

Statistical analysis was performed using GraphPrism version 7 software. Statistical significances were determined using one or two-way analysis of variance with multiple comparisons (ANOVA). The level of statistical significance is represented as follows: ns = *P* > 0.05, *P ≤ 0.05, **P ≤ 0.01, ***P ≤ 0.001 and ****P ≤ 0.0001.

## Results

### A DSF screening cascade to identify HER3-binding compounds

**Figure 1.**
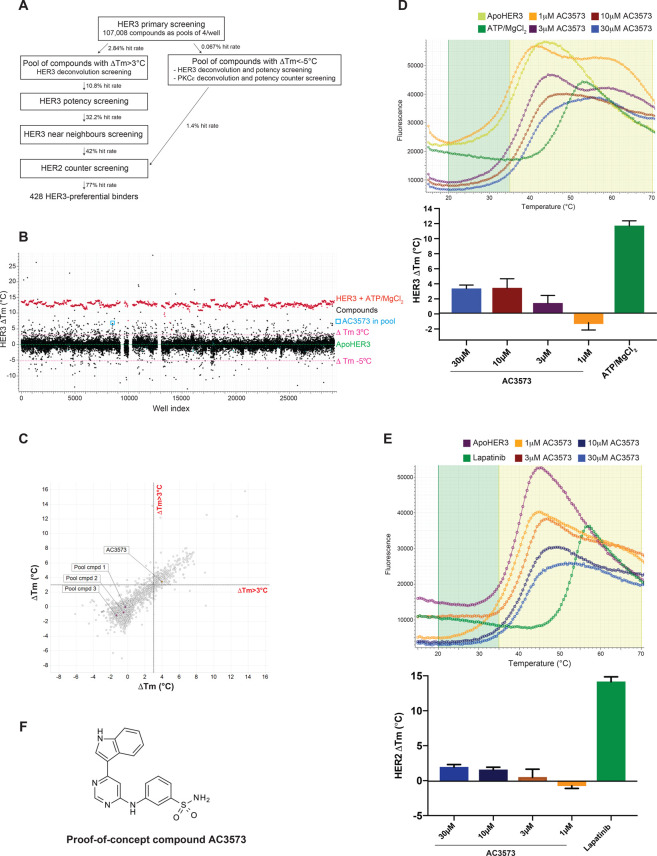
A differential scanning fluorimetry (DSF) assay compound screen identifies 428 HER3-preferential binders from a 107 008 compound library. (**A**) Overview of the screening strategy and hit rate for each step of the screening cascade. (**B**) Scatter graph of DSF primary screening shows pools of compounds that either stabilise or destabilise HER3 kinase domain *in vitro*. ApoHER3 (HER3 kinase domain with no ligand) was used as a control for all DSF analyses (mean of ApoHER3 *T*_m_ across the plates set at Δ*T*_m_ = 0°C is indicated by the dash green line) and Δ*T*_m_ values were calculated for each pool of compounds tested at 30 μM each (*N *= 1). Cut-off values of Δ*T*_m_>3°C and Δ*T*_m_ < −5°C were used to designate hits (shown as dashed pink lines). The pool of compounds containing proof-of-concept compound AC3573 is circled. Gaps indicate the rare plate failures (three plates). Compounds from these plates were retested during the deconvolution screen. (**C**) Correlation of shift in HER3 *T*_m_ value induced by deconvoluted pool compounds tested at 30 μM between the two repeats of deconvolution screening. AC3573 and its three pool compounds are highlighted. (**D**) Dose-dependent analysis of thermal shifts induced by AC3573 compound tested at 1, 3, 10 and 30 μM in the HER3 potency screen. The change in HER3 *T*_m_ value (Δ*T*_m_), compared with ApoHER3 control, is reported as mean ± SD from *N *= 4 independent experiments. Representative thermal denaturation profiles of recombinant HER3 kinase domain, in the presence of 0.3% DMSO (Apo control), ATP/MgCl2 (ATP control) or AC3573 tested at 1, 3, 10 and 30 μM. (E) Dose-dependent analysis of thermal shifts induced by AC3573 compound tested at 1, 3, 10 and 30 μM in the HER2 counter screen. The change in HER2 *T*_m_ value (Δ*T*_m_), compared with ApoHER2 control (HER2 recombinant protein without ligand), is reported as mean ± SD from *N *= 3 independent experiments. A representative plot of the counter screening thermal denaturation profiles of recombinant HER2 kinase domain, in the presence of 0.3% DMSO (Apo control), 1 μM lapatinib (Lapatinib control) or AC3573 compound tested at 1, 3, 10 and 30 μM is shown. (**F**) Chemical structure of the proof-of-concept compound AC3573 identified in the DSF screen.

To identify novel HER3 specific binding compounds from a compound library, we designed and validated a multiplex medium-throughput thermal shift assay, established on a 384-well microtiter platform, with a focus on assay reproducibility (RZ factor over 0.8 for all the assays performed at all the screening steps) and sensitivity (Figure 1A and Supplementary Table S1). Thermal shift assays measure protein thermostability which can be influenced by compound binding [52]. This can result either in a destabilising effect, conferring a decrease in thermostability (lower melting temperature *T*_m_) or a stabilising effect, which leads to an increase in thermostability and to a higher *T*_m_. Pilot tests were performed to fine-tune assay methodologies, including recombinant protein concentration, positive and negative controls generating a suitable assay window (Supplementary Figure S1A), melting curve protocols and whether known HER3-binding compounds could be identified from pools of compounds and then validated at a subsequent compound deconvolution stage (Supplementary Figure S1B).

Having established a robust screening platform, a total of 107 008 small molecule compounds were screened as mixtures of four compounds per well (a total of 26 752 wells) at 30 μM each in a primary library screen. Intraplate controls for data normalisation consisted of 1.2% DMSO as a neutral control and 200 μM ATP/10 mM MgCl2 as a positive control. We applied a cut-off value of Δ*T*_m_ > 3°C (3×SD of 1.2% DMSO neutral controls, which fell above the noise of the assay) to define hit pools of compounds that had the ability to stabilise HER3, and a cut-off of Δ*T*_m_ < −5°C to select hit pools of compounds that could destabilise HER3 (Figure 1B). In the primary screen, valid data were obtained for 25 285 wells, 709 of which induced a shift in HER3 *T*_m_ over 3°C, corresponding to 2835 compounds and 2.84% hit rate (Figure 1A,B, Supplementary Figure S2A for proof-of-concept compound AC3573 data). In the second step of screening, hit pools of compounds were arrayed as individual compounds and 3119 compounds (2835 hits plus 284 compounds from failed wells from the primary screen) were assessed at 30 μM, using the same thermal shift assay settings as for the primary screen (0.3% DMSO as neutral controls reflecting the single compound use, 200 μM ATP/10 mM MgCl2 as positive controls) applying again a cut-off value of Δ*T*_m_ > 3°C (Figure 1C). From this deconvolution, 338 hit compounds were confirmed as stabilising HER3 in the two rounds of deconvolution screening performed. As an exemplar, the proof-of-concept compound, AC3573 (see further below), was identified and validated as being the only compound from its pool to be responsible for the shift induced in HER3 *T*_m_ (Figure 1C and Supplementary Figure S2B).

The few pools of compounds which induced negative thermal shifts (Δ*T*_m_ < −5°C), compared with the control ApoHER3, implying they had a destabilising effect on HER3, were deconvoluted to 72 individual compounds which were tested for their binding to HER3 in a dose–response assay. To assess whether these compounds would specifically destabilise HER3 or have generic destabilising properties, they were also counter screened against another kinase, PKC*ε*. From those 72 compounds, only one compound showed a reproducible destabilising activity towards HER3, but was found to induce a negative shift in PKC*ε T*_m_ also, suggesting this compound's destabilising effect was not specific to HER3 (data not shown). This was confirmed, as in the HER2 counter screen, this compound induced a negative shift in the HER2 *T*_m_.

Hit compounds stabilising HER3 were then evaluated for their relative binding affinity in a dose–response thermal shift assay at 1, 3, 10 and 30 μM (Supplementary Figure S2C). After duplicate confirmation, 109 compounds were shown to induce a stabilisation of at least 3°C of the HER3 *T*_m_ at or below 10 μM. The proof-of-concept compound was confirmed as a HER3 binder inducing a shift in HER3 *T*_m_ of over 3°C with the dose–response indicating an estimated IC50 of 4 μM (Figure 1D and Supplementary Table S2). Near neighbours of those 109 hits were selected and 663 new compounds were screened in duplicate for their relative potency at 1, 3, 10 and 30 μM by thermal shift assay. From those, 446 compounds passed the selection criteria, i.e. induced shift in HER3 *T*_m _> 3°C below 10 μM (data not shown).

We sought to identify compounds binding specifically to HER3. Since HER2 and HER3 are highly homologous EGFR family members [53], we took forward the 555 compounds identified as stabilising HER3, to be counter screened for HER2 binding. Compounds were assayed at 1, 3, 10 and 30 μM by DSF, using 0.3% DMSO as a neutral control and 1 μM lapatinib as a positive control. Compounds binding to HER2, i.e. inducing a shift in HER2 *T*_m_ over 2°C (3×SD) compared with the DMSO neutral control were disregarded (Supplementary Figure S2D). This counter screening resulted in 428 compounds preferentially binding to HER3, corresponding to an overall hit rate of 0.4% from the initial library. Proof-of-concept compound AC3573 was identified as a HER3-preferential binder, since it did not induce a shift in HER2 *T*_m_ over the 2°C cut-off, even at the 30 μM highest concentration (Figure 1E, Supplementary Figure S2D and Supplementary Table S2). AC3573 chemical structure revealed it bore an amino-pyrimidine scaffold, suggesting it could interact with the HER3 ATP-binding site (Figure 1F).

### Proof-of-concept compound AC3573 abrogates HER2–HER3 signalling in cells and is more specific for HER3

**Figure 2.**
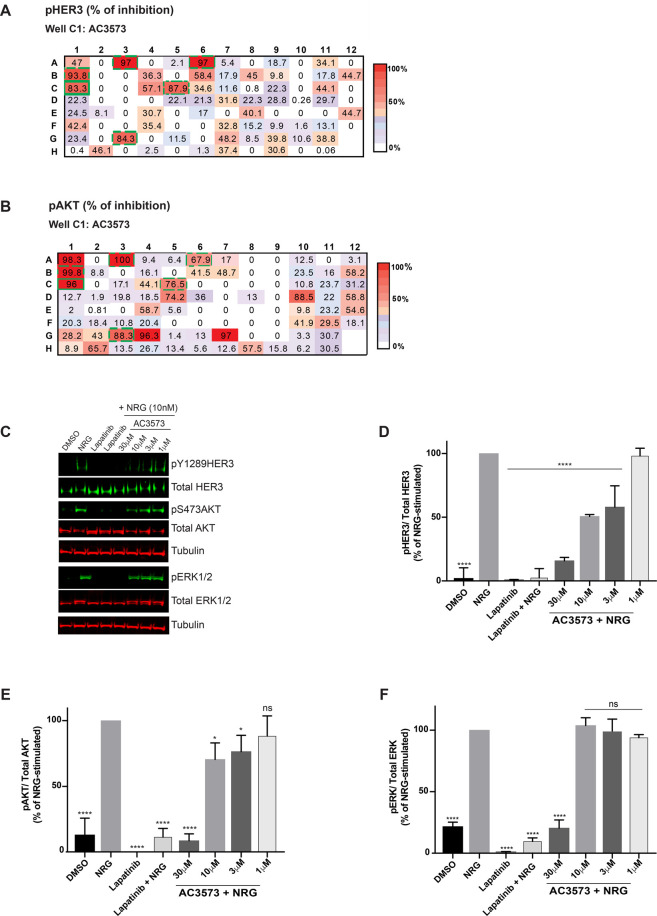
AC3573 compound inhibits heregulin-induced signalling. (**A**,**B**) Effects of the selected 94 representative compounds of each cluster on heregulin-induced signalling in a cell-based screen. SK-BR-3 cells were serum-starved for 16 h and treated for 1 h with either DMSO or 30 μM of each compound and subjected to 15 min NRG stimulation (10 nM). After lysis, whole-cell extracts were immunoblotted with phospho-HER3 (Tyr1289), Total HER3, phospho-Akt (Ser473) or total Akt primary antibodies. pHER3/Total HER3 and pAkt/Total Akt ratios were quantified relative to the DMSO NRG-stimulated condition using ImageStudioLite software and normalised to *α*-tubulin. Heat maps illustrate and summarise quantitative Western Blot results as a percentage of inhibition of HER3 (**A**) and Akt (**B**) phosphorylation for each compound. Data are means from *N *= 2 independent experiments. The proof-of-concept compound AC3573 corresponds to well C1 (full line green box, 83.3% inhibition of pHER3 and 96% inhibition of pAKT). The five other hit compounds are indicated by dashed line green boxes (compounds A3, A6, B1, C5 and G3). (**C**) SK-BR-3 cells were serum-starved for 16 h and treated for 1 h with either DMSO, the indicated concentrations of AC3573 or 1 μM lapatinib and subjected or not to 15 min NRG stimulation (10 nM). After lysis, whole-cell extracts were immunoblotted with the indicated primary antibodies for assessment of effects of AC3573 on NRG-induced signalling. Data shown are representative of three independent experiments. (**D**) pHER3Tyr1289/Total HER3 ratio was quantified relative to NRG-stimulated using ImageStudioLite software. Blots were normalised to *α*-tubulin. Data are means ± SD from *N *= 3 independent experiments. (**E**) Quantification of pAktSer473/Total Akt ratio as percentage of NRG-stimulated. Data are means ± SD from *N *= 3 independent experiments. (**F**) Quantification of pERK1/2/Total ERK ratio as percentage of NRG-stimulated. Data are means ± SD from *N *= 3 independent experiments.

The initially confirmed HER3-binding compounds reflected 108 chemical clusters and, by disregarding clusters comprising singletons and clusters of compounds whose near neighbours were identified as binding to HER2 in the counter screen, 94 remained of interest. The best representatives of each cluster, and those with good predicted permeability, were selected for screening in cell-based assays. The HER2–HER3 heterodimer is a potent mitogenic and survival unit, in particular through the recruitment of the PI3K-AKT pathway in HER2-dependent breast cancer [17]. So, we assessed the ability of the representative compounds of each cluster to affect HER2–HER3 downstream signalling in a model HER2-dependent breast cancer cell line, SK-BR-3. Ninety-four compounds were tested at 30 μM in SK-BR-3 cells for their ability to inhibit heregulin-induced HER3 phosphorylation and downstream signalling (Akt activation, Figure 2A,B). Of those 94 compounds, six were found to strongly inhibit HER3 phosphorylation in response to NRG stimulation (>80% inhibition compared with NRG alone) and to affect NRG-induced HER2–HER3 downstream Akt activation (>65% inhibition compared with NRG alone) (Figure 2A,B, proof-of-concept compound AC3573 and compounds A3, A6, B1, C5 and G3). These six compounds, including the proof-of-concept compound AC3573, were then assessed in a dose–response assay for their ability to prevent HER2–HER3 activation in response to NRG stimulation. SK-BR-3 cells were treated for 1h with 1, 3, 10 and 30 μM compounds or with 1 μM lapatinib as a positive control, stimulated for 15 min with NRG and assessed by Western Blotting for NRG-induced HER3 phosphorylation and downstream signalling. As expected, lapatinib alone or as a pre-treatment prior to NRG stimulation, inhibited all signalling from the HER2–HER3 dimer (Figure 2C). The AC3573 compound (Figure 2C), and four others (Supplementary Figure S3A–C) were confirmed as inhibiting NRG-induced HER3 phosphorylation (>80% inhibition compared with NRG-stimulated at 30 μM and 50% inhibition at 10 μM, Figure 2D) and HER3 downstream signalling (>80% inhibition of Akt and ERK phosphorylation as compared with NRG only treated controls at 30 μM, Figure 2E,F).

Since the readout for HER3 function in cells required HER2 activity, it was critical to confirm HER2 inactivity of these compounds. Although these six compounds did not test positive in the HER2 thermal shift assay, suggesting that they were not HER2 binders, we nevertheless evaluated their ability to inhibit HER2 using an *in vitro* kinase assay (ThermoFisher). Unexpectedly, all except the proof-of-concept compound AC3573, were active against HER2 (IC50 values ranging from 0.54 to 1.13 μM, Supplementary Table S3 and >10 μM for AC3573 assayed at the *K*_m_ for ATP, Supplementary Figure S4). A more extensive *in vitro* profiling against a panel of 400 kinases was performed with 1 μM AC3573 (see Supplementary Table S4), which did not show any significant activity on any other of the EGFR family members and confirmed its lack of activity on HER2 (49% mean inhibition on EGFR, 14% on HER4 and 3% on HER2).

**Figure 3.**
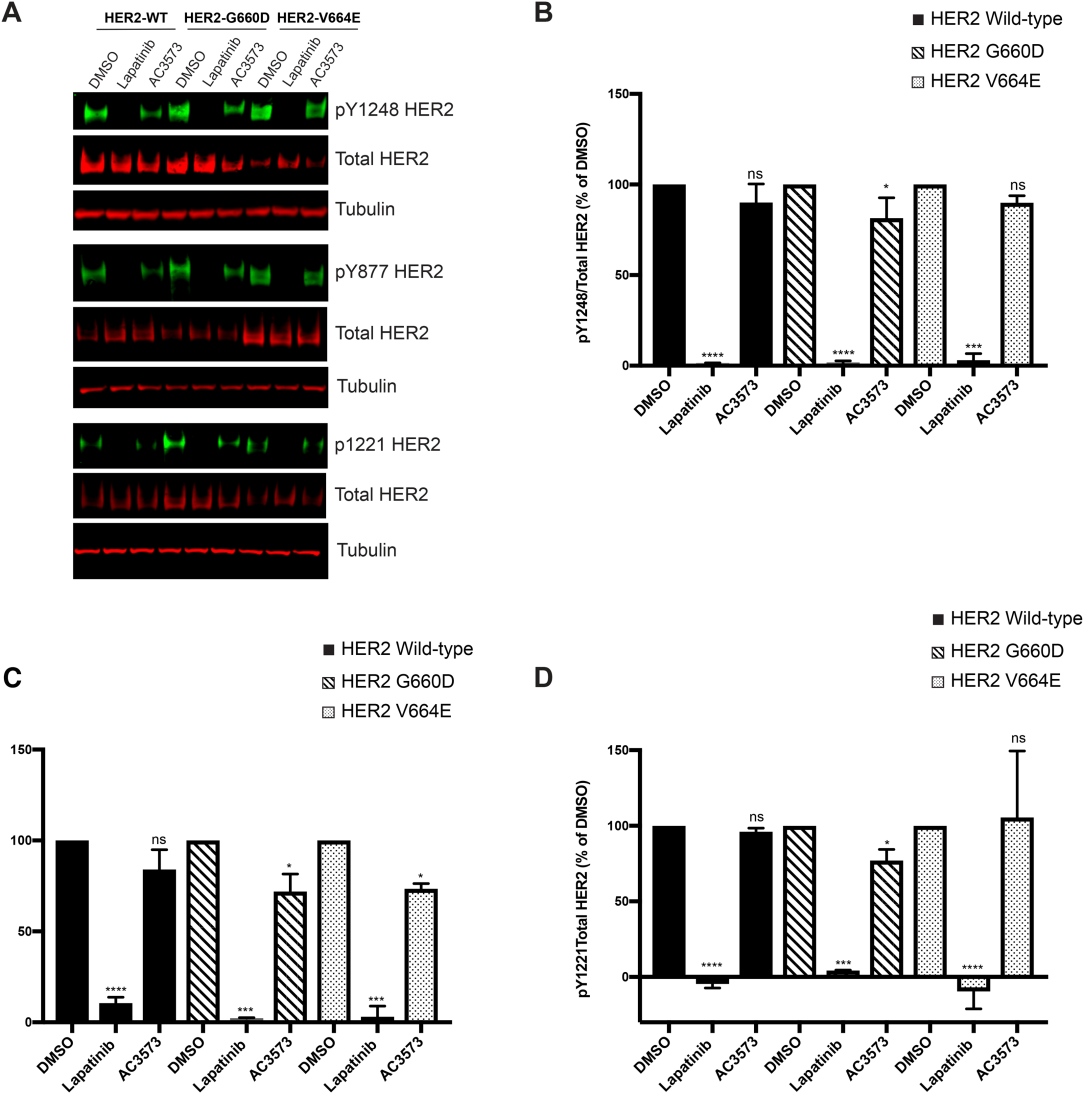
AC3573 compound is more specific for HER3 and has no activity towards HER2. (**A**) CHO cells were transfected with expression plasmids encoding the indicated HER2 full length wild-type (WT) or oncogenic point mutants G600D or V664E. Forty hours post-transfection cells were treated for 1 h with 1 μM lapatinib or 30 μM AC3573 and HER2 phosphorylation status was assessed by Western blot with the indicated primary antibodies. (**B**) Quantification of pY1248HER2/Total HER2 ratio as percentage of DMSO treated conditions. Data are means ± SD from *N *= 3 independent experiments. (**C**) Quantification of pY877HER2/Total HER2 ratio as percentage of DMSO treated conditions. Data are means ± SD from *N *= 3 independent experiments. (**D**) Quantification of pY1221HER2/Total HER2 ratio as percentage of DMSO treated conditions. Data are means ± SD from *N *= 3 independent experiments.

To confirm the lack of AC3573 activity against HER2, we assessed its effects on HER2 in an activated ‘HER2 only’ dependent cellular context. HER2 full length wild-type or oncogenic point mutants G660D and V664E were transiently expressed in CHO cells and we evaluated the effects of AC3573 at 30 μM on HER2 autonomous activation, assessing HER2 autophosphorylation status on Y1248, Y877 and Y1221/22 by Western blot (Figure 3A). As expected 1 μM lapatinib strongly impaired HER2 autophosphorylation on those three autophosphorylation sites for all the HER2 overexpressed forms, whereas 30 μM AC3573 had no or little effect on HER2 autonomous activation (Figure 3B–D), either for HER2 wild-type or for HER2 oncogenic mutants, indicating that the inhibition of NRG-induced HER2–HER3 signalling is not via HER2.

### Mechanistic analysis of AC3573 binding to HER3

To gain insight into the mechanism of action of AC3573, we sought to determine the X-ray crystal structure of AC3573 bound to the HER3 kinase domain. The sparse matrix screens used were JCSG core suite screens (Hampton Research), comprising 384 crystallisation formulations, Index (Hampton Research) and PACT (NeXtal). Further screens exploring various precipitant and pHs, including AmSO4 (NeXtal), COMPAS (NeXtal), MPD (NeXtal) and Salt-RX (Hampton Research) were also used to find initial crystallisation hits. None of the conditions yielded promising hits, but resulted mainly in a high degree of precipitation. These findings contrast with the success reported for crystallisation with bosutinib (see https://www.rcsb.org/structure/6OP9), consistent with the observations that AC3573 acts differently.

**Figure 4.**
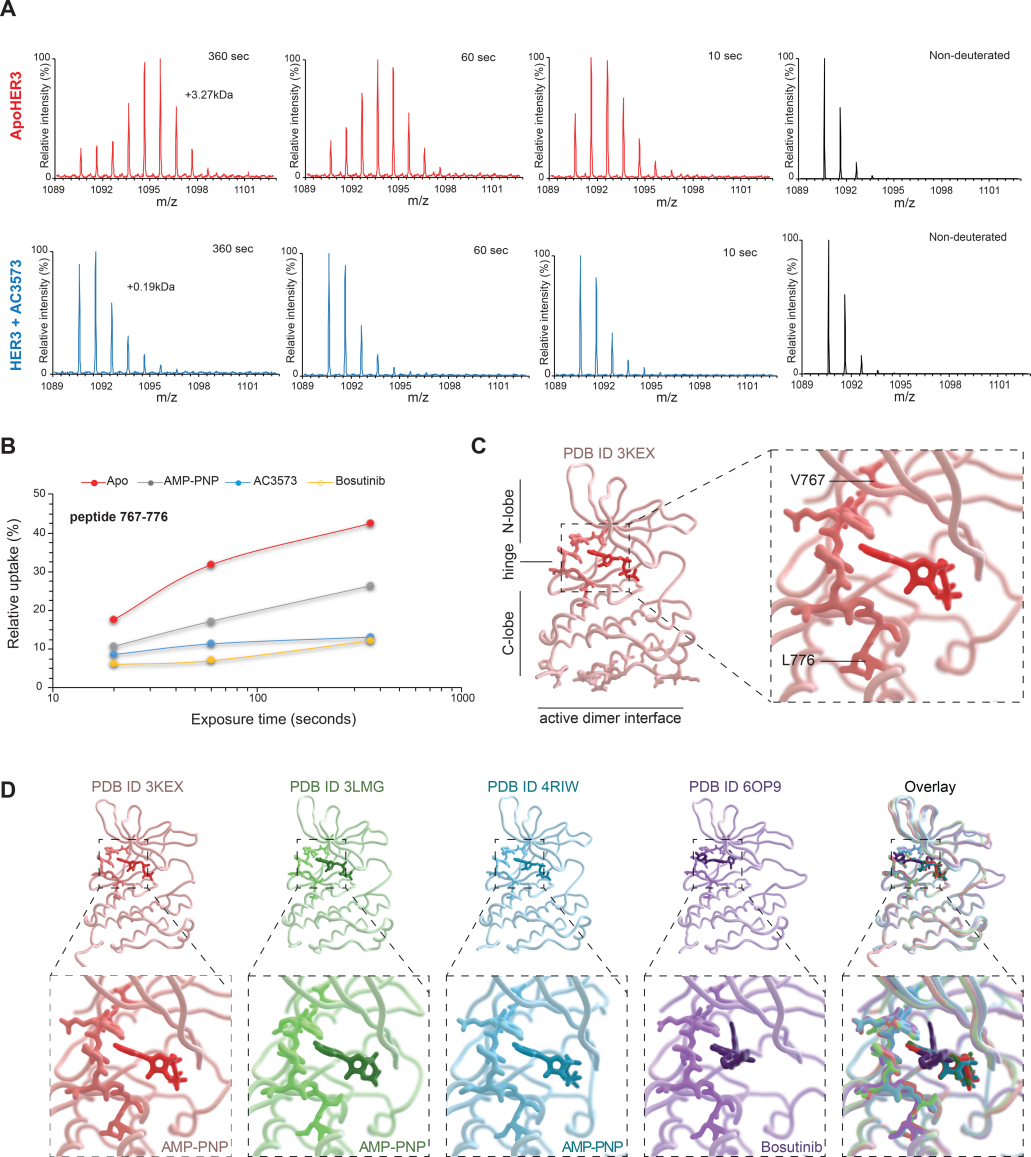
HDX-MS identified HER3 nucleotide-binding pocket region as AC3573 binding site. (**A**) Differences in deuterium incorporation between ApoHER3 (HER3 no ligand) and HER3 in the presence of AC3573. Mass spectra of the peptide 767–776 for ApoHER3 (left, in red) and HER3 + AC3573 at 100 μM (right, in blue) are shown, as non-deuterated and for deuterium labelling times of 10 s, 1 min and 6 min. Levels of deuteration are determined through comparison of the average mass from the intensity-weighted centroid *m*/*z* value of the peptide. The mass spectra show that the peptide 767–776 has an increase in mass of 3.27 kDa following deuterium incorporation for ApoHER3, compared with an increase in 0.19 kDa for HER3 incubated with AC3573. (**B**) The deuterium uptake (as relative uptake in %), resolved for peptide 767–776, plotted at various time points for ApoHER3 and HER3 incubated with 100 μM of AMP-PNP, bosutinib or AC3573. Deuterated sample data were obtained in triplicate for all three deuterium uptake time points. The protection of the peptide comprising residues 767–776 suggests AMP-PNP, bosutinib and AC3573 have the same binding site to HER3. (**C**) Left: crystal structure of HER3 bound to AMP-PNP (PDB:3KEX), showing AC3573 compound binding site (highlighted). Right: close up of AC3573 binding site (highlighted), corresponding to peptide 767–776 VTQYLPGSL, encompassing the hinge region. (**D**) Individual structures of HER3 tyrosine kinase domain (amino acids 684–1020) bound to AMP-PNP (PDB:3KEX and PDB:3LMG), to bosutinib (PDB:6OP9) and in the context of EGFR/HER3 kinase domain heterodimer (PDB:4RIW) and structural alignments (overlay) of these four crystal structures showing that AC3573 binding site (highlighted as heavier backbone chain in each structure) is the hinge peptide capping the nucleotide-binding pocket.

In the absence of direct structural insight, we turned to HDX-MS to identify the AC3573 binding site in HER3. HER3 pepsin-digests provided a good sequence coverage (over 95%, Supplementary Figure S5). HDX-MS experiments were carried out at five time points of exchange (10, 30, 60, 360 and 1800 s) in the absence of any ligand (ApoHER3) and presence of 100 μM AC3573 (HER3 + AC3573). The only observed differences in deuteration were specific to the peptide 767–776, with AC3573 resulting in protection against deuterium exchange relative to ApoHER3 (Figure 4A).

We compared the rate of deuterium exchange in the presence of AC3573 to two other known HER3 binders, the ATP analogue AMP-PNP [53] and the ATP-competitive inhibitor bosutinib [41]. HDX-MS revealed that AC3573, AMP-PNP and bosutinib bound to the same site of HER3, peptide 767–776, corresponding to the hinge region between the N-and C-lobes [53] (Figure 4B–C, Supplementary Movie 1). This suggests a similar mode of binding of AC3573 and these other HER3 ligands, as shown in Figure 4D.

**Figure 5.**
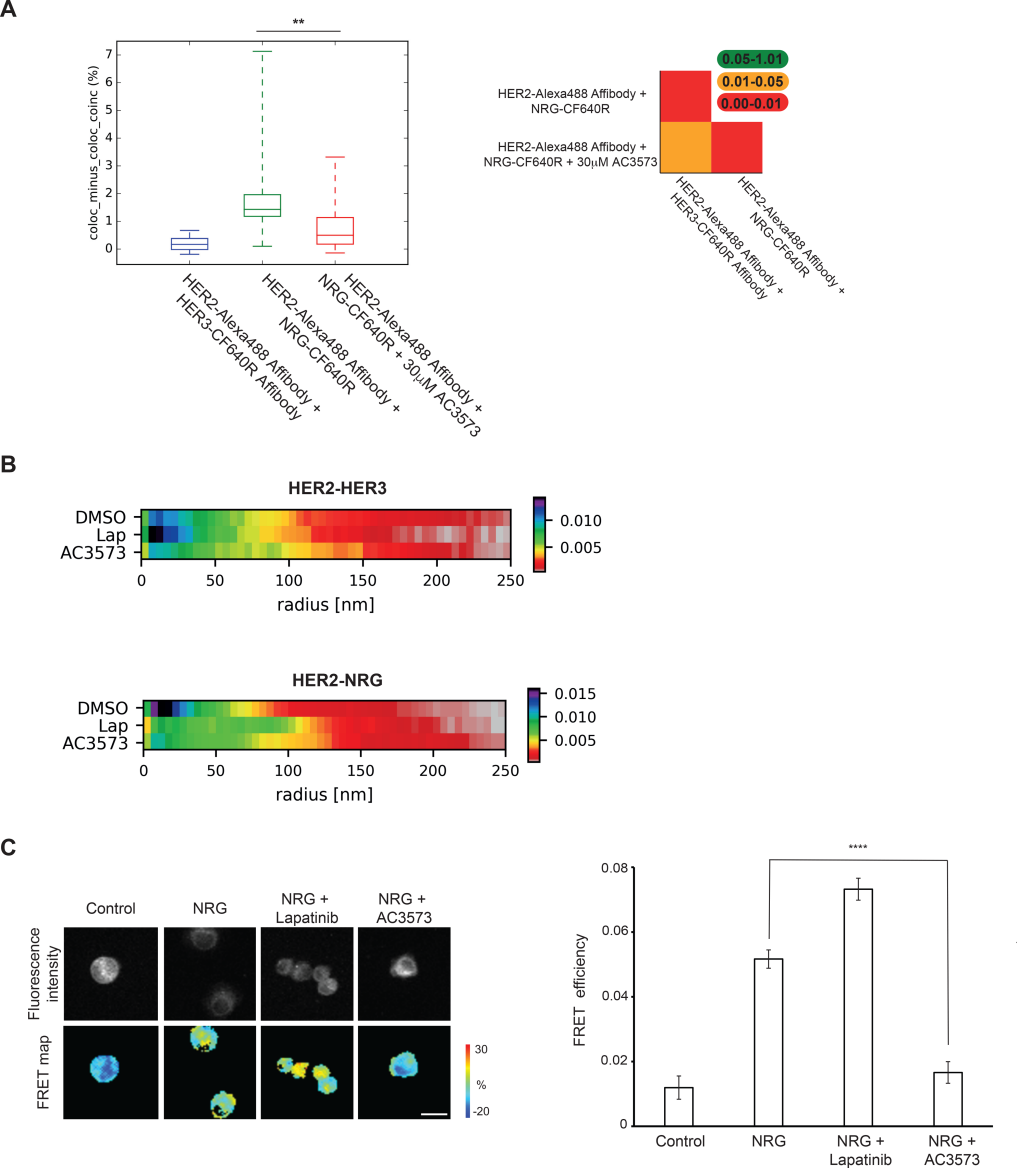
AC3573 compound abrogates the formation of the active HER2–HER3 heterodimer. (**A**) Left: two-colour single particle tracking (SPT) on live CHO cells showing the fraction of tracks where two HER2 and HER3 molecules labelled with Alexa 488-Affibody and CF640R SE-Affibody or NRG-CF640R, respectively, spent at least five 50 ms frames together within <1 pixel (pairwise particle colocalisation fraction) for cells treated or not with 30 μM AC3573. Right: significance of differences between compared conditions in two-colour SPT. The plots show tables from the two-tailed Kolmogorov–Smirnov test *P*-value. (**B**) Heat map of the probability of the distance of nearest HER2 neighbour of HER3. Cluster measurements from STORM data taken from SK-BR-3 cells labelled with HER2-Alexa488 Affibody and HER3-CF640R SE Affibody (HER2–HER3) or NRG-CF640R SE (HER2-NRG) ± 1 μM lapatinib or 30 μM AC3573 compound. (**C**) AC3573 blocks NRG-induced dimerisation of HER2 and HER3 receptors in SK-BR-3 breast cancer cells. Cells were seeded on glass coverslips and 72 h later serum-starved for additional 24 h before treatment with AC3573 (30 μM) or lapatinib (1 μM) for 1 h. NRG was added at 10 nM for 15 min. Cells were fixed and stained with anti-HER2-Cy5 and anti-HER3-Alexa-fluor-546 and fluorescence lifetime images were acquired on an open-microscope system. The inhibitory effect of AC3573 is statistically significant (NRG vs NRG + AC3573) with *P *< 0.0001. The difference between untreated (control) and NRG or lapatinib-treated cells is statistically significant too (*P *< 0.0001). Scale bar is 25 μm.

In view of the inhibitory nucleotide pocket occupation by AC3573 and the pro-function and pro-complex formation of other occupants (i.e. ATP and the ATP-competitive inhibitor bosutinib [39]), we investigated how the AC3573 compound impacted HER2–HER3 dimer formation using multiple approaches. Initially, we used two-colour single particle tracking, a method previously used to report the colocalisation fraction of pairwise receptor complexes [50]. HER2 and HER3 wild-type full length receptors were overexpressed in CHO cells and labelled with affibodies to define their basal state. Stimulated states of the receptors were assessed by labelling with HER2-Alexa488 Affibody and NRG-CF640R, which resulted in a significant increase in colocalisation between HER2 and HER3 receptors (mean of colocalisation 0.20% in the basal state and 1.71% in the NRG-stimulated state) (Figure 5A). HER2–HER3 heterocomplex formation was impaired by pre-treatment with 30 μM AC3573, as shown by the significant decrease in colocalisation frequency compared with NRG-stimulated only cells (mean of colocalisation 0.79% vs 1.71% in the NRG-state, *P*-value < 0.01, Figure 5A right panel). Interaction durations *τ*ON, calculated over the colocalization events detected for each condition, show that the stability of the activated complex does not change significantly in the presence or absence of AC3573 (data not shown).

We then used stochastic optical reconstruction microscopy (STORM) to assess the effects of AC3573 on endogenous HER2 and HER3 clustering in SK-BR-3 cells. STORM data, acquired from the equatorial region of dual labelled cells, is presented as the probability of a HER2 receptor localisation at nearest neighbour distances from a HER3 receptor (normalised for all neighbours at a distance of <300 nm) for active and inactive HER3 in untreated or lapatinib or AC3573-treated cells. This data showed that AC3573, unlike lapatinib, has no effect on basal HER2, which is consistent with its preferential targeting of HER3 (Figure 5B and Supplementary Figure S6A, HER2–HER3 conditions). Moreover, AC3573 compound did not appear to induce HER3 oligomerisation, as HER3 cluster size and the number of HER3 molecules per cluster did not differ between DMSO and AC3573 only treated cells (Supplementary Figure S6B).

As expected, NRG stimulation yielded the greatest probability of close range (i.e. <50 nm) HER2 detection with HER3 (Figure 5B and Supplementary Figure S6A, HER2-NRG conditions). The most frequent nearest neighbour distance occurs at ∼15 nm indicating that directly interacting HER2–HER3 heterodimers are most likely. Furthermore, STORM data validated observations from our single particle tracking, since AC3573 treatment of SK-BR-3 cells showed a reduced probability of direct interactions between NRG-bound HER3 and HER2, as the distance between near neighbour molecules increased with AC3573, suggesting a disruption of HER2–HER3 association. Lapatinib unexpectedly also induced a distancing of HER2–HER3 molecules, however, there were different patterns of distribution of near neighbours between AC3573 and lapatinib. As shown in the plot of the differences in the probability of nearest neighbour distances, HER3–HER2 separations were significantly different between the treatment conditions (Supplementary Figure S6A). In particular, for NRG- bound HER3, HER2 receptors clustered at distances of <50 nm in DMSO treated cells, at ∼80–100 nm in lapatinib-treated cells or at ∼200 nm in AC3573-treated cells.

To address the AC3573 mechanism of action further, we used FLIM-FRET to assess AC3573 effects on HER2–HER3 endogenous dimer formation in SK-BR-3 cells. As expected, NRG-induced HER2 and HER3 dimerisation, which is enhanced by pre-treatment with lapatinib, as we showed previously ([39], Figure 5C). In contrast, we found that NRG-driven HER2–HER3 heterodimerisation was strongly blocked by pre-treatment with AC3573, as FRET efficiency was decreased to levels comparable to the unstimulated condition. These results confirmed the data obtained with STORM and suggested that AC3573 binding to HER3 could induce conformational changes preventing the formation of productive complexes with HER2. Our data, therefore, indicate a neutralising effect of AC3573, since it reduces active HER3–HER2 heterocomplexes to levels observed for inactive HER3–HER2 heterocomplexes.

## Discussion

Pseudokinases have an impaired kinase domain and primarily signal through non-catalytic mechanisms. However, like their catalytically active counterparts, these unusual kinases are of importance for normal physiological processes, and many pseudokinases contribute to the pathology of human diseases, including cancer [54]. For example, signalling by the pseudokinase HER3 underlies resistance to EGFR and HER2-targeted therapies (discussed in [55]).

In catalytically competent kinases, a wide spectrum of conformational states is achieved by the kinase domain and those transitions from ‘off’ to ‘on’ states are not only indispensable for the catalysis of phosphorylation, but as well for their allosteric functions [56]. These transitions, from inactive to active state, can be influenced by small molecules stabilising conformations which modulate the catalytic and allosteric functions of kinases.

There are similarities between pseudokinase regulation and that of their active counterparts. Pseudokinases can indeed adopt conformations that are reminiscent of the on/off state of active kinases, and those conformational changes are critical for their role as allosteric regulators of other proteins’ activities. Hence, disrupting a functional conformation of the pseudokinase domain by small molecules, to impinge on their allosteric activator role, could be an interesting therapeutic strategy in targeting pseudokinases, which have been linked to many pathologies, but against which treatments remain limited.

The pseudokinase HER3 has been associated with cancer progression, but so far, none of the HER3-targeted therapies tested in clinical trials has shown greater efficacy than existing treatments [9]. Thereby there is desire for alternative treatment modalities aimed at HER3. As an exemplar to validate the concept of allosteric inhibitors of pseudokinases, we sought to identify HER3 allosteric inhibitors abrogating productive HER2–HER3 dimer formation by: (i) screening for compounds binding to HER3 *in vitro* that do not impact on HER2 *in vitro* using a thermal shift assay approach: (ii) identifying that subset of compounds that inhibit NRG-induced HER2–HER3 signalling in cells; (iii) defining the hit compounds mechanism of action. To uncover such compounds, we designed and validated a two-step DSF screening strategy. We first screened a multiplex compound library against HER3 binding, then deconvoluted the pools of compounds which induced positive thermal shifts, compared with the control ApoHER3, consistent with a stabilising effect of HER3. Compounds potency was finally evaluated by dose–response thermal shift assays, and the most potent compounds, showing a binding activity towards HER3 at or below 10 μM, were selected. To discover specific HER3-binding compounds, in the second step of the screening cascade, we counter screened the most potent compounds against HER2 binding, filtering out compounds and their near neighbours that bound to HER2. This led to the identification of 428 small molecules of multiple chemical classes preferentially binding to HER3, including predicted non-hinge binder and hinge-binder compounds, which, based on their Structure-Activity-Relationship, fitted into 94 chemical clusters of interest.

To discriminate compounds binding to HER3 and stabilising it in a conformation unfavourable to HER2–HER3 dimer formation, we determined the ability of the best representative compounds of each cluster to inhibit HER3-dependent activity. HER3 does not have measurable activity towards substrates *in vitro* [53], despite retaining a weak autophosphorylation activity *in vitro* under specific circumstances [42]. Hence, to assess its engagement by the hit compounds, we screened HER3-binding, HER2-non-binding compounds, in a cell-based assay for their efficacy in abrogating HER3-dependent functions. Using the HER2 positive breast cancer model SK-BR-3 cells, we identified a few compounds which robustly blocked NRG-induced HER3 phosphorylation and HER2–HER3 dimer downstream signalling, including Akt and ERK phosphorylation. In this proof-of-concept study, we applied a stringent cut-off of 80% of inhibition of phosphorylation to select compounds interfering with HER2–HER3-dependent signalling. However, in a drug discovery context, hits with a weaker effect in cells might afford interesting chemotypes to take forward through medicinal chemistry.

We subsequently focused our mechanism of action study on the proof-of-concept compound AC3573. The relative specificity of AC3573 to HER3 was confirmed by *in vitro* profiling against a panel of kinases, including the three other members of the EGFR family, and its lack of activity, against HER2 in particular, was corroborated in a ‘HER2 only’ dependent cellular context, where AC3573 had no effect on HER2 autophosphorylation. AC3573's HER3-dependent mechanism was confirmed by single particle tracking in CHO cells overexpressing HER2 and HER3 and by clustering data on endogenous receptors in SK-BR-3 cells, as unlike lapatinib, AC3573 did not have any effects on the basal state of HER2. It was determined that NRG-induced HER2–HER3 signalling inhibition by AC3573 was due to its ability to disrupt HER2–HER3 dimer formation, as clustering and single particle tracking showed that AC3573 decreased HER2–HER3 colocalisation induced by NRG and FLIM-FRET data showed that AC3573 disrupted HER2–HER3 dimer formation. Our data suggest that AC3573 may exert HER3-dependent pharmacology by abrogating HER3 function as an allosteric activator of HER2. Whether it acts to lock HER3 in an inactive conformation has yet to be elucidated, but structural studies of HER3 in complex with AC3573 will most likely resolve this.

AC3573 contains an amino-pyrimidine moiety and HDX-MS experiments confirmed AC3573 as a nucleotide-pocket occupier. Such molecules have been shown to interfere with pseudokinase functions. For example, TYK2 signalling is blocked by ATP-competitive compounds binding to its pseudokinase domain and locking it in a conformation that stabilises an autoinhibitory interaction with its catalytic kinase domain [57]. For HER3, we recently showed that ATP-binding site non-occupation compromises HER2–HER3 complex formation and signalling, but that the ATP-competitive inhibitor, bosutinib, enhances HER3 allosteric activator function [39]. Hence, we did not expect other ATP-site binders to be inhibitory and to disrupt HER2–HER3 dimer formation. However, it seems that HER3 nucleotide-binding site occupation by bosutinib or AC3573 lead to different functional states of the receptor. Unlike bosutinib, AC3573 either promotes a conformation of HER3 preventing its interaction with HER2 to generate signalling competent complexes or perhaps achieves this outcome through some steric effect on dimer formation. However, it is noted that the dimer interface is some distance from the ATP-binding pocket (see Figure 4C, lower panel), so the latter scenario seems less likely as a mechanism of action. Diversifying hits with non-ATP-site HER3 binders may require exploration of libraries excluding such scaffolds.

In summary, we identified a small molecule that binds more specifically to HER3 than any other EGFR family member or the 400 kinases tested. This HER3-binding compound is able to disrupt NRG-induced HER2–HER3 complex formation, resulting in the inhibition of oncogenic downstream signalling in SK-BR-3 breast cancer cells. Our data suggest that AC3573 may exert HER3-dependent pharmacology abrogating HER3 function as an allosteric activator of HER2. This proof-of-concept study has implications not only for HER3 interventions, but also for the identification and/or design of small molecules that can perturb the biological function of other pseudokinases.

## Open Access

Open access for this article was enabled by the participation of The Francis Crick Institute in an all-inclusive *Read & Publish* pilot with Portland Press and the Biochemical Society under a transformative agreement with JISC.

## Abbreviations

CHO: Chinese Hamster Ovary
DSF: differential scanning fluorimetry
EGFR: epidermal growth factor receptor
FBS: fetal bovine serum
HDX-MS: hydrogen-deuterium exchange mass spectrometry
STORM: stochastic optical reconstruction microscopy
