# Supplementary material for: A small molecule inhibitor of HER3: a proof-of-concept study
Source: Biochem J. 2020 Sep 10;477(17):3329–47. doi: 10.1042/BCJ20200496 (PMC7489893; doi:10.1042/BCJ20200496)
Supplement: Supplementary Figures S1-S6 and Tables S1-S4 [file BCJ-477-3329-s1.pdf]

Supplementary Figure 1

A

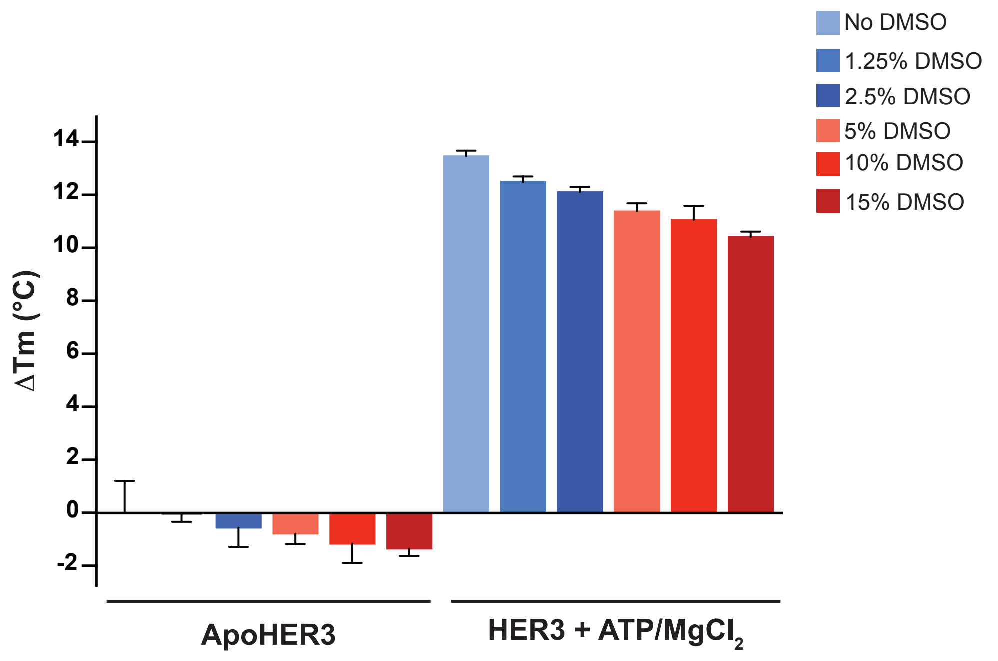

B

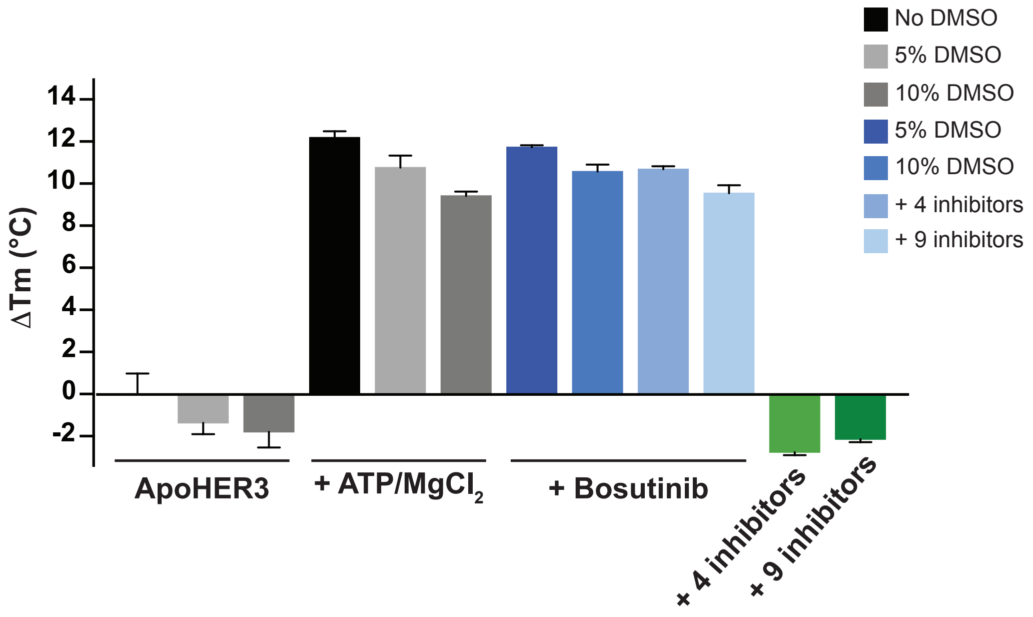

Supplementary Figure 2

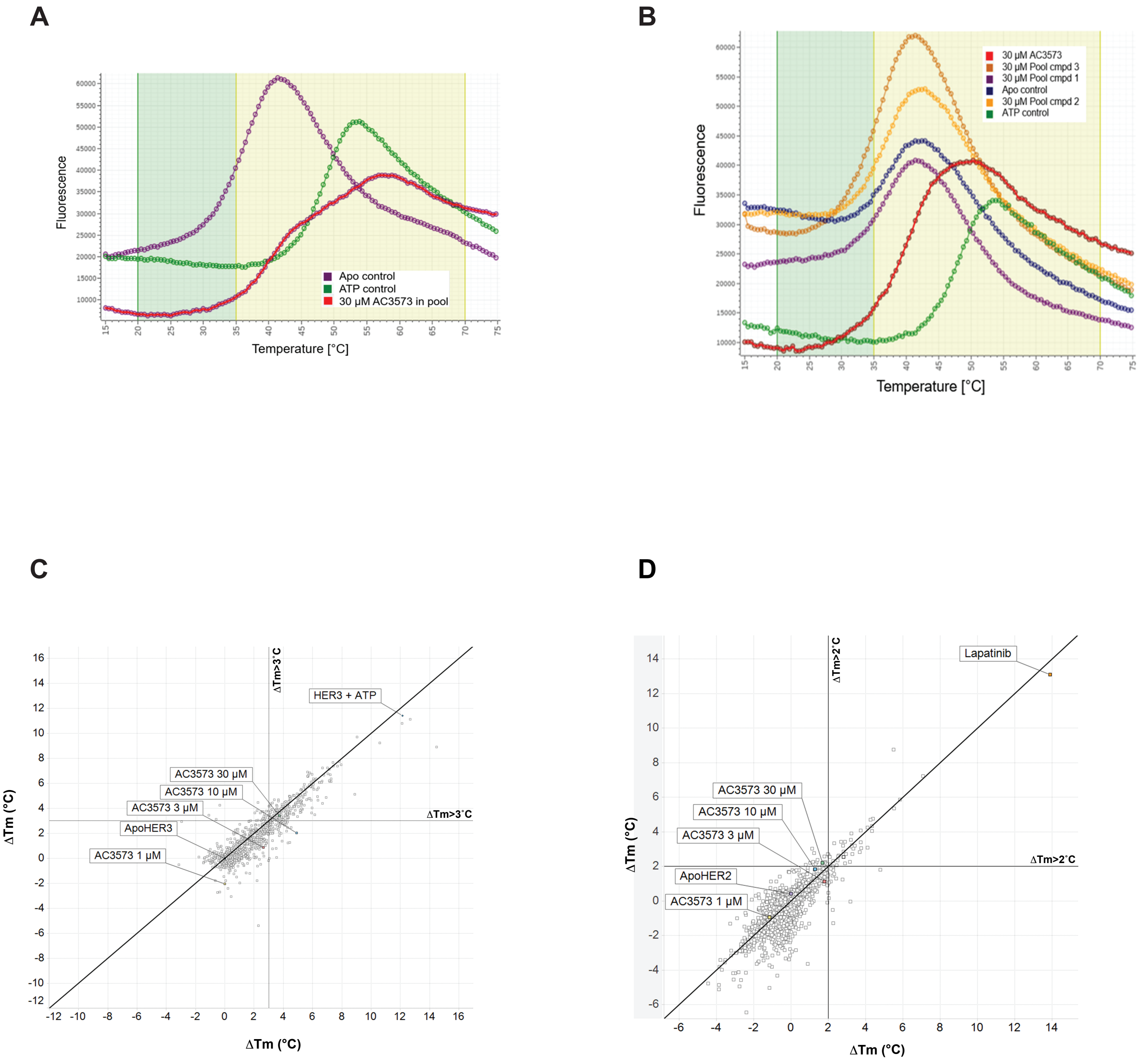

Supplementary Figure 3

A

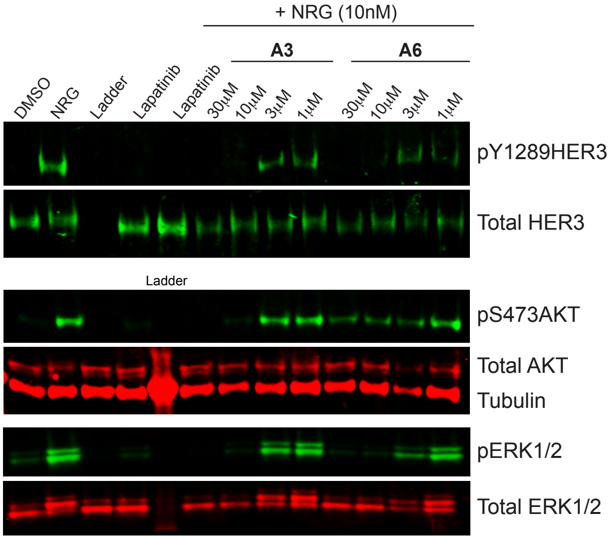

B

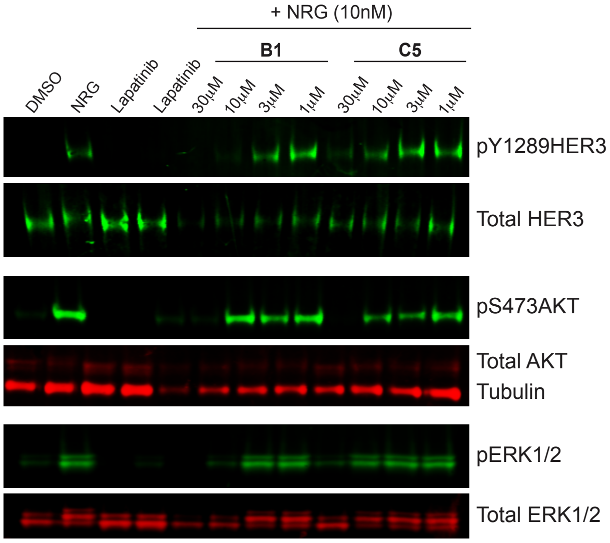

C

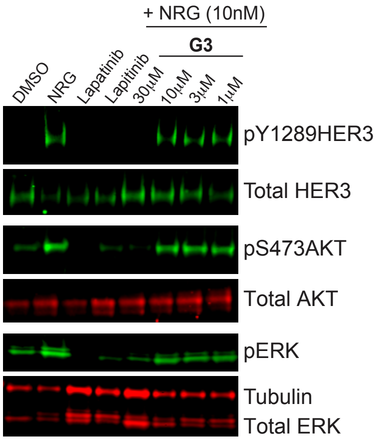

Supplementary Figure 4

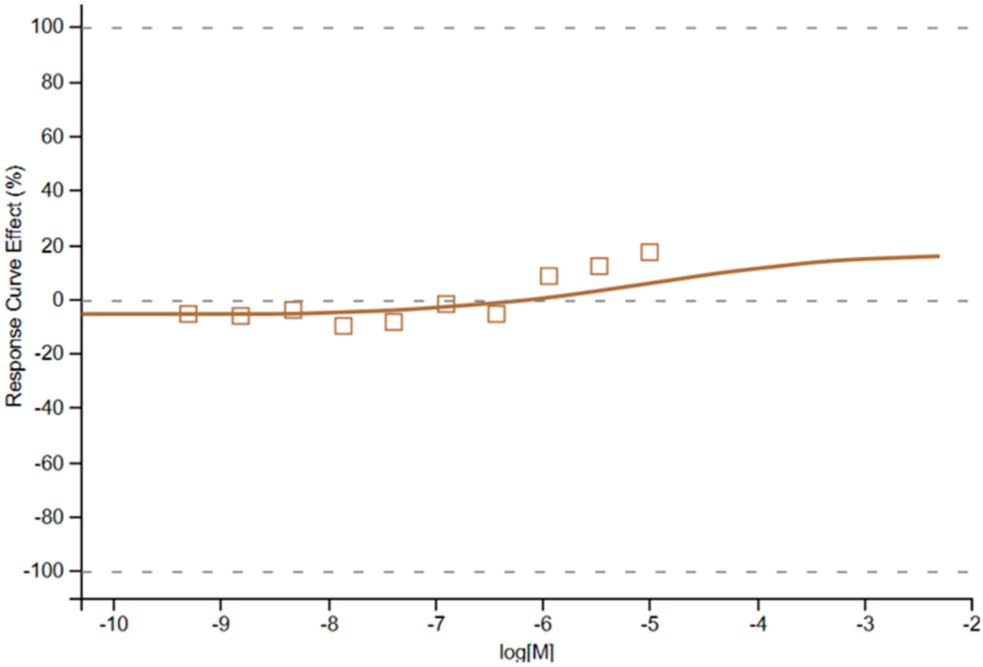

Supplementary Figure 5

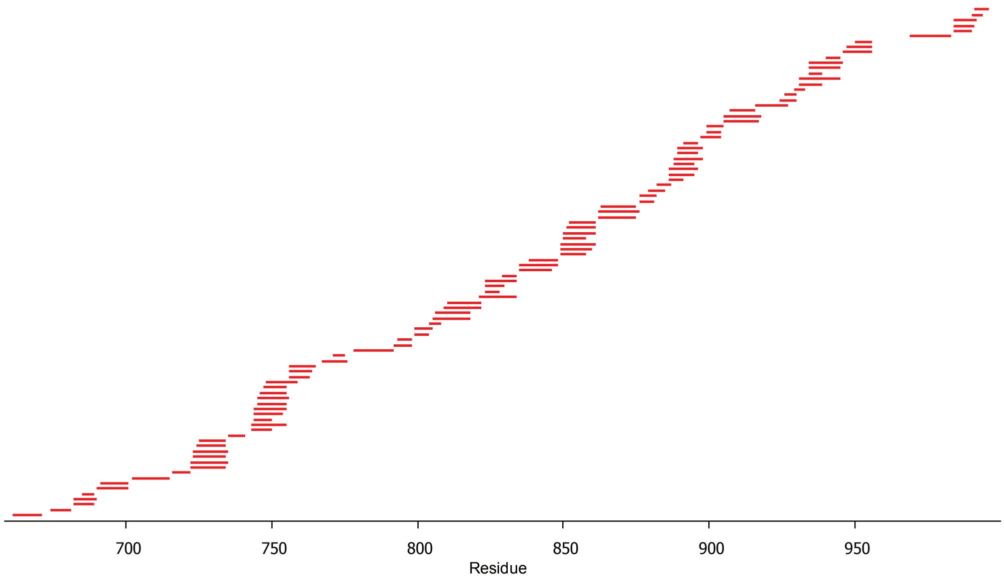

Supplementary Figure 6

A

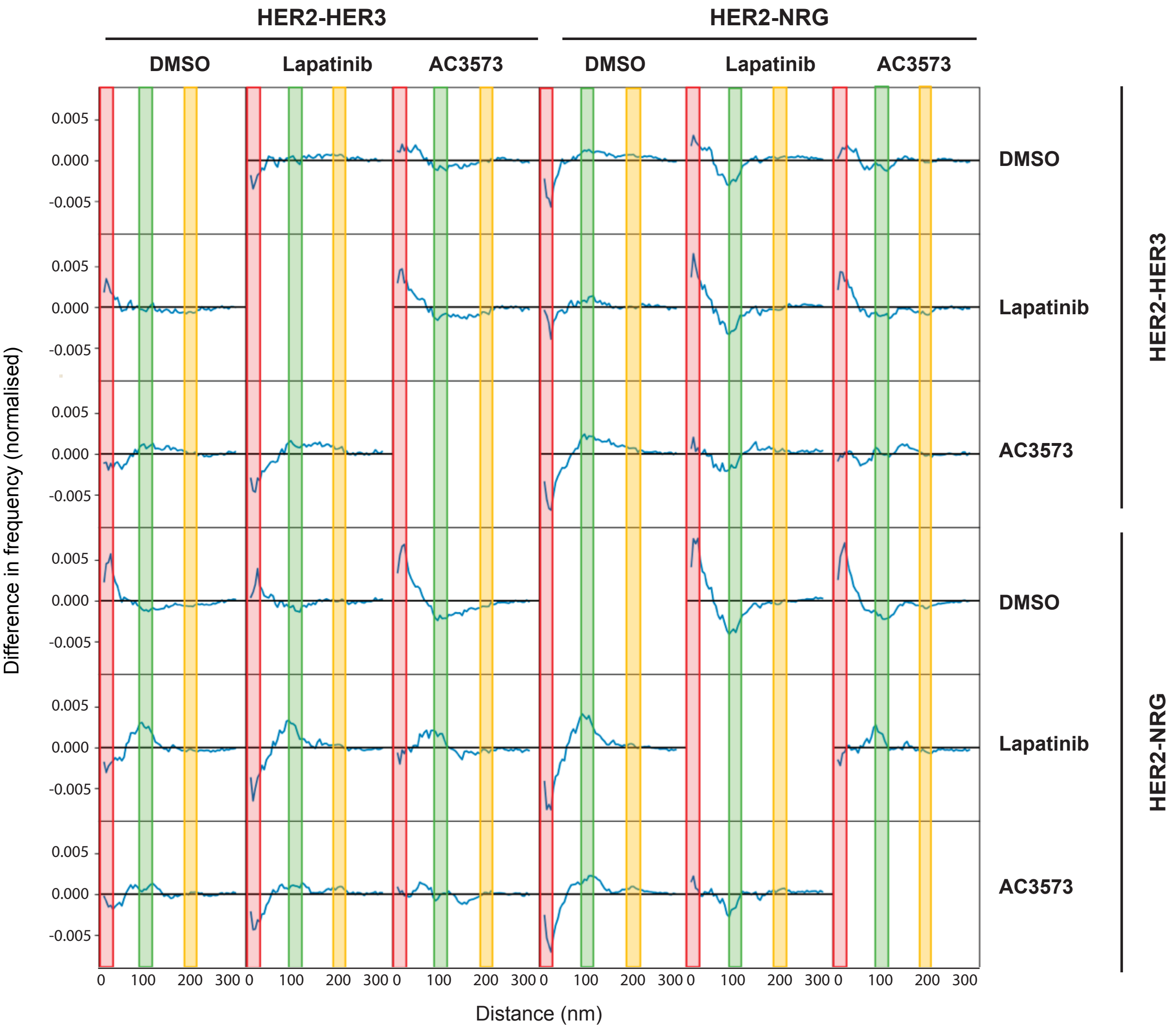

B

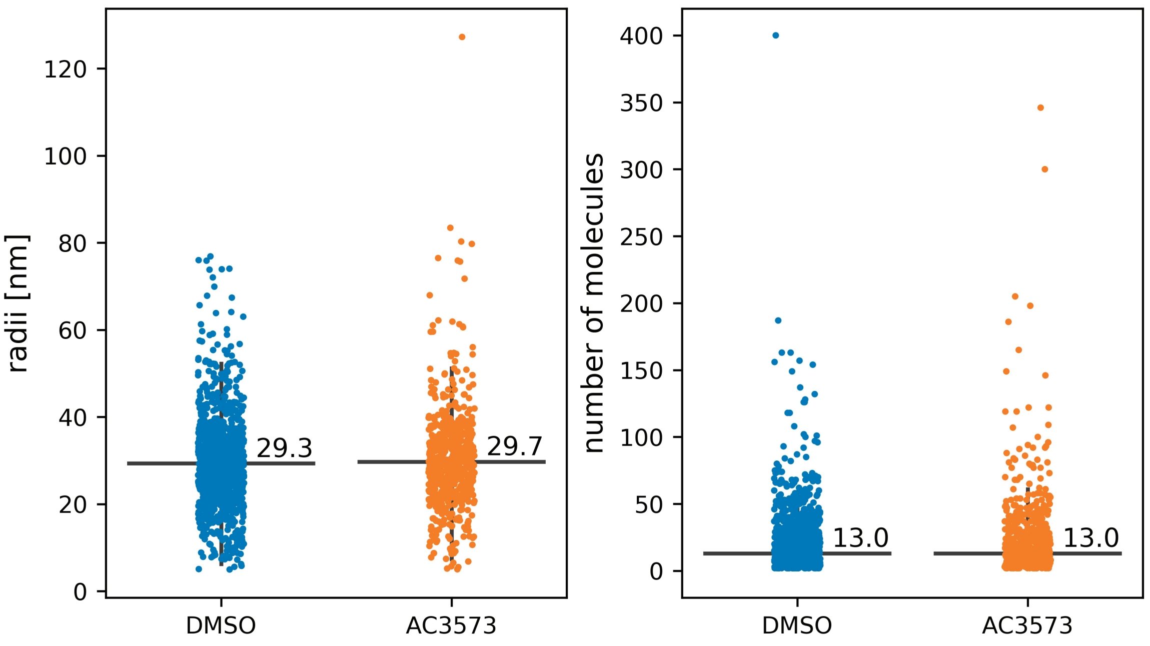

Supplementary Table 1

	Experimental design	Compounds	Hit selection	Controls (Tm (°C) ± SD)	RZ factor
**Stage I - HER3 screening cascade**					
Step 1 - Primary screening	Pools of 4 compounds/ well at 30μM each, n=1	107,008	ΔTm>3°C	TmApo 34.71 ± 0.44<br>TmATP 47.75 ± 0.33	0.88
Step 2 - Deconvolution screening	Deconvoluted compounds at 30μM, n=2	3,119	ΔTm>3°C	TmApo 35.60 ± 0.45<br>TmATP 47.46 ± 0.28	0.88
Step 3 - Potency screening	Dose-response at 30, 10, 3, 1μM, n=2	338	ΔTm>3°C and potency<10μM	TmApo 35.09 ± 1.56<br>TmATP 47.63 ± 0.76	0.87
Step 4 - Near neighbours screening	Dose-response at 30, 10, 3, 1μM, n=2	663	ΔTm>3°C	TmApo 33.47 ± 0.58<br>TmATP 48.45 ± 0.29	0.83
**Stage II - HER2 counter screening**	Dose-response at 30, 10, 3, 1μM, n=2	555	ΔTm>2°C	TmApo 38.64 ± 0.31<br>TmLapatinib 52.67 ± 0.28	0.93

Supplementary Table 2

	Concentration, μM	ΔTm (°C) ± SD
**HER3 primary screen, pools of 4 compounds**	30 each	6.6
**HER3 deconvolution screen**	30	3.65 ± 0.25
**HER3 potency screen**	30	3.2 ± 0.24
	10	3.28 ± 0.62
	3	1.27 ± 0.51
	1	-1.34 ± 0.48
**HER2 counter screen**	30	1.95 ± 0.25
	10	1.56 ± 0.26
	3	0.48 ± 0.52
	1	-0.75 ± 0.18

Supplementary Table 3

Compound ID	Activity against HER2 , IC_{50} (μM)
C5	0.54
B1	0.84
A3	0.88
A6	1.13
AC3573	>10

Supplementary Table 4

Kinase Name	Mean Inhibition (%)
AAK1	54
ABL1	73
ABL2 (Arg)	67
ACVR1 (ALK2)	12
ACVR1B (ALK4)	6
ACVR2A	1
ACVR2B	15
ACVRL1 (ALK1)	7
ADCK3	-1
ADRBK1 (GRK2)	-2
ADRBK2 (GRK3)	-4
AKT1 (PKB alpha)	4
AKT2 (PKB beta)	1
AKT3 (PKB gamma)	19
ALK	5
AMPK (A1/B1/G2)	2
AMPK (A1/B1/G3)	7
AMPK (A1/B2/G1)	7
AMPK (A1/B2/G2)	8
AMPK (A1/B2/G3)	5
AMPK (A2/B1/G2)	6
AMPK (A2/B1/G3)	5
AMPK (A2/B2/G1)	10
AMPK (A2/B2/G2)	0
AMPK (A2/B2/G3)	8
AMPK A1/B1/G1	-8
AMPK A2/B1/G1	4
ANKK1	15
AURKA (Aurora A)	5
AURKB (Aurora B)	2
AURKC (Aurora C)	9
AXL	4
BLK	34
BMPR1A (ALK3)	2
BMPR1B (ALK6)	7
BMPR2	54
BMX	12
BRAF	7
BRSK1 (SAD1)	22
BRSK2	-10
BTK	13
CAMK1 (CaMK1)	-20

CAMK1G (CAMKI gamma)	6
CAMK2A (CaMKII alpha)	9
CAMK2B (CaMKII beta)	-3
CAMK2D (CaMKII delta)	11
CAMK2G (CaMKII gamma)	-2
CAMK4 (CaMKIV)	3
CAMKK1 (CAMKKA)	6
CAMKK2 (CaMKK beta)	30
CASK	-3
CDC42 BPA (MRCKA)	3
CDC42 BPB (MRCKB)	2
CDC42 BPG (MRCKG)	1
CDC7/DBF4	23
CDK1/cyclin B	15
CDK11 (Inactive)	10
CDK11/cyclin C	7
CDK13/cyclin K	20
CDK14 (PFTK1)/cyclin Y	-5
CDK16 (PCTK1)/cyclin Y	7
CDK17/cyclin Y	5
CDK18/cyclin Y	4
CDK2/cyclin A	20
CDK2/cyclin A1	36
CDK2/cyclin E1	-8
CDK3/cyclin E1	17
CDK4/cyclin D1	12
CDK4/cyclin D3	1
CDK5 (Inactive)	17
CDK5/p25	12
CDK5/p35	15
CDK6/cyclin D1	6
CDK7/cyclin H/MNAT1	7
CDK8/cyclin C	7
CDK9 (Inactive)	65
CDK9/cyclin K	54
CDK9/cyclin T1	83
CDKL5	11
CHEK1 (CHK1)	4
CHEK2 (CHK2)	1
CHUK (IKK alpha)	1
CLK1	3
CLK2	18
CLK3	8
CLK4	30
CSF1R (FMS)	47
CSK	1
CSNK1A1 (CK1 alpha 1)	3
CSNK1A1L	2

CSNK1D (CK1 delta)	10
CSNK1E (CK1 epsilon)	5
CSNK1G1 (CK1 gamma 1)	-2
CSNK1G2 (CK1 gamma 2)	6
CSNK1G3 (CK1 gamma 3)	7
CSNK2A1 (CK2 alpha 1)	20
CSNK2A2 (CK2 alpha 2)	32
CSNK2A2 (CK2 alpha 2)	32
DAPK1	2
DAPK2	5
DAPK3 (ZIPK)	1
DCAMKL1 (DCLK1)	3
DCAMKL2 (DCK2)	10
DDR1	10
DDR2	0
DDR2	-3
DMPK	2
DNA-PK	37
DYRK1A	19
DYRK1B	10
DYRK2	79
DYRK3	27
DYRK4	17
EEF2K	4
EGFR (ErbB1)	49
EIF2AK2 (PKR)	3
EPHA1	38
EPHA2	21
EPHA3	-12
EPHA4	10
EPHA5	0
EPHA5	14
EPHA6	6
EPHA7	-3
EPHA8	6
EPHB1	21
EPHB2	14
EPHB3	8
EPHB4	8
ERBB2 (HER2)	3
ERBB4 (HER4)	14
ERN1	-15
ERN2	-3
FER	9
FES (FPS)	4
FGFR1	5
FGFR1 V561M	9
FGFR2	2

FGFR3	-4
FGFR4	-4
FGR	47
FLT1 (VEGFR1)	-3
FLT3	24
FLT4 (VEGFR3)	-2
FRAP1 (mTOR)	11
FRK (PTK5)	7
FYN	10
FYN A	38
GAK	58
GRK1	6
GRK4	-25
GRK5	-4
GRK6	-18
GRK7	3
GSG2 (Haspin)	6
GSK3A (GSK3 alpha)	5
GSK3B (GSK3 beta)	12
HCK	39
HIPK1 (Myak)	0
HIPK2	8
HIPK3 (YAK1)	9
HIPK4	8
HUNK	21
ICK	5
IGF1R	2
IKBKB (IKK beta)	-3
IKBKE (IKK epsilon)	1
INSR	3
INSRR (IRR)	-3
IRAK1	62
IRAK3	48
IRAK4	14
ITK	1
JAK1	10
JAK2	-1
JAK3	-4
KDR (VEGFR2)	8
KIT	1
KSR2	8
LATS2	17
LCK	27
LIMK1	40
LIMK2	33
LRRK2	29
LRRK2 FL	35
LTK (TYK1)	-1

LYN A	28
LYN B	61
MAP2K1 (MEK1)	-5
MAP2K2 (MEK2)	5
MAP2K4 (MEK4)	14
MAP2K5 (MEK5)	20
MAP2K6 (MKK6)	4
MAP2K6 (MKK6)	-1
MAP3K10 (MLK2)	26
MAP3K11 (MLK3)	10
MAP3K14 (NIK)	-4
MAP3K19 (YSK4)	8
MAP3K2 (MEKK2)	-18
MAP3K3 (MEKK3)	-21
MAP3K5 (ASK1)	-26
MAP3K7/MAP3K7IP1 (TAK1-TAB1)	18
MAP3K8 (COT)	11
MAP3K9 (MLK1)	13
MAP4K1 (HPK1)	10
MAP4K2 (GCK)	17
MAP4K3 (GLK)	9
MAP4K4 (HGK)	63
MAP4K5 (KHS1)	-8
MAPK1 (ERK2)	11
MAPK10 (JNK3)	11
MAPK11 (p38 beta)	11
MAPK12 (p38 gamma)	10
MAPK13 (p38 delta)	8
MAPK14 (p38 alpha)	4
MAPK15 (ERK7)	84
MAPK3 (ERK1)	12
MAPK7 (ERK5)	3
MAPK8 (JNK1)	17
MAPK9 (JNK2)	2
MAPKAPK2	6
MAPKAPK3	7
MAPKAPK5 (PRAK)	4
MARK1 (MARK)	6
MARK2	14
MARK3	4
MARK4	-7
MASTL	-4
MATK (HYL)	8
MELK	36
MERTK (cMER)	3
MET (cMet)	8
MINK1	50
MKNK1 (MNK1)	41

MKNK2 (MNK2)	63
MKNK2 (MNK2)	57
MLCK (MLCK2)	26
MLK4	31
MST1R (RON)	-1
MST4	7
MUSK	-2
MYLK (MLCK)	3
MYLK2 (skMLCK)	0
MYLK4	50
MYO3A (MYO3 alpha)	9
MYO3B (MYO3 beta)	13
NEK1	7
NEK2	-6
NEK2	2
NEK4	10
NEK6	5
NEK8	3
NEK9	-2
NIM1K	8
NLK	16
NTRK1 (TRKA)	6
NTRK2 (TRKB)	7
NTRK3 (TRKC)	3
NUAK1 (ARK5)	24
NUAK2	29
PAK1	9
PAK2 (PAK65)	10
PAK3	1
PAK4	6
PAK6	11
PAK7 (KIAA1264)	1
PASK	5
PDGFRA (PDGFR alpha)	13
PDGFRB (PDGFR beta)	11
PDK1	8
PEAK1	9
PHKG1	11
PHKG2	1
PI4K2A (PI4K2 alpha)	3
PI4K2B (PI4K2 beta)	-2
PI4KA (PI4K alpha)	10
PI4KB (PI4K beta)	57
PIK3C2A (PI3K-C2 alpha)	6
PIK3C2B (PI3K-C2 beta)	5
PIK3C2G (PI3K-C2 gamma)	17
PIK3C3 (hVPS34)	1
PIK3CA/PIK3R1 (p110 alpha/p85 alpha)	10

PIK3CA/PIK3R3 (p110 alpha/p55 gamma)	30
PIK3CB/PIK3R1 (p110 beta/p85 alpha)	-3
PIK3CB/PIK3R2 (p110 beta/p85 beta)	0
PIK3CD/PIK3R1 (p110 delta/p85 alpha)	-6
PIK3CG (p110 gamma)	20
PIM1	37
PIM2	5
PIM3	-7
PIP4K2A	79
PIP5K1A	48
PIP5K1B	93
PIP5K1C	91
PKMYT1	15
PKN1 (PRK1)	3
PKN2 (PRK2)	9
PLK1	6
PLK2	-2
PLK3	-22
PLK4	26
PRKACA (PKA)	0
PRKACB (PRKAC beta)	12
PRKACG (PRKAC gamma)	2
PRKCA (PKC alpha)	2
PRKCB1 (PKC beta I)	1
PRKCB2 (PKC beta II)	16
PRKCD (PKC delta)	-5
PRKCE (PKC epsilon)	-2
PRKCG (PKC gamma)	0
PRKCH (PKC eta)	11
PRKCI (PKC iota)	-16
PRKCN (PKD3)	20
PRKCQ (PKC theta)	11
PRKCZ (PKC zeta)	14
PRKD1 (PKC mu)	31
PRKD2 (PKD2)	8
PRKG1	4
PRKG2 (PKG2)	5
PRKX	10
PTK2 (FAK)	9
PTK2B (FAK2)	14
PTK6 (Brk)	73
RAF1 (cRAF) Y340D Y341D	4
RET	6
RIPK2	17
RIPK3	46
ROCK1	-4
ROCK2	-7

ROS1	18
RPS6KA1 (RSK1)	8
RPS6KA2 (RSK3)	28
RPS6KA3 (RSK2)	8
RPS6KA4 (MSK2)	14
RPS6KA5 (MSK1)	7
RPS6KA6 (RSK4)	35
RPS6KB1 (p70S6K)	3
RPS6KB2 (p70S6Kb)	1
SBK1	6
SGK (SGK1)	5
SGK (SGK1)	3
SGK2	6
SGKL (SGK3)	6
SIK1	32
SIK3	24
SLK	5
SNF1LK2	13
SPHK1	9
SPHK2	-6
SRC	23
SRMS (Srm)	27
SRPK1	7
SRPK2	-6
STK16 (PKL12)	-2
STK17A (DRAK1)	30
STK17B (DRAK2)	47
STK22B (TSSK2)	5
STK22D (TSSK1)	13
STK23 (MSSK1)	4
STK24 (MST3)	6
STK25 (YSK1)	7
STK3 (MST2)	8
STK32B (YANK2)	1
STK32C (YANK3)	-1
STK33	18
STK38 (NDR)	15
STK38L (NDR2)	-22
STK39 (STLK3)	6
STK4 (MST1)	1
SYK	11
TAOK1	18
TAOK2 (TAO1)	4
TAOK3 (JIK)	-5
TBK1	11
TEC	4
TEK (Tie2)	8
TESK1	6

TESK2	-4
TGFBR1 (ALK5)	2
TGFBR2	44
TLK1	0
TLK2	31
TNIK	47
TNK1	17
TNK2 (ACK)	48
TTK	16
TXK	25
TYK2	1
TYRO3 (RSE)	25
ULK1	-1
ULK2	6
ULK3	15
VRK2	14
WEE1	34
WNK1	5
WNK2	8
WNK3	4
YES1	30
ZAK	5
ZAP70	-5

Supplementary Figures and Tables Legends

Supplementary Figure 1

Differential scanning fluorimetry multiplex screening set up.

A) Signal window and DMSO tolerance of the thermal shift assay. Recombinant HER3 kinase domain in absence of any ligand (ApoHER3) was used as a neutral control and recombinant HER3 kinase domain in the presence of 200µM ATP/10mM $MgCl_2$ (ATP/$MgCl_2$) was used as positive control. Assays were performed with an increasing percentage of DMSO. Data showed a large signal window between the neutral and positive controls (shift in HER3 Tm of 13.5°C without DMSO) and which was not significantly reduced at the DMSO concentration used in the screening (shift in HER3 Tm of 12.5°C between ApoHER3 and HER3 + ATP/$MgCl_2$ at 1.25% DMSO). **B)** Thermal shift binding assay of recombinant HER3 kinase domain with selected kinase inhibitors. Recombinant HER3 kinase domain was assayed in absence of any ligand (ApoHER3), or in presence of 200µM ATP/10mM $MgCl_2$ (ATP/$MgCl_2$) or in presence of 1µM of bosutinib alone or in combination with a mixture of 4 other inhibitors at 1µM each (BLU577, MG-132, LY294002, CRT6854) or in combination with a mixture of 9 other inhibitors at 1µM each (BLU577, BIM1, BX-912, LY294002, staurosporine, MG-132, bortezomib, MG-132, CRT0066854). Data showed that HER3-binding compounds, e.g. bosutinib, could be identified from pools of compounds, as the shift in HER3 Tm induced by bosutinib was not affected as a result of pooling compounds.

Supplementary Figure 2

Differential scanning fluorimetry data obtained for the proof-of-concept compound AC3573 at all the steps of the screening cascade.

A) Primary screening thermal denaturation profiles of recombinant HER3 kinase domain, in the presence of 1.2% DMSO (ApoHER3 control, in the absence of any ligand), ATP/$MgCl_2$ (ATP control) and the pool of 4 compounds containing AC3573. Compounds were tested at 30µM each. **B)** Deconvolution screening thermal denaturation profiles of recombinant HER3 kinase domain, in presence of 0.3% DMSO (ApoHER3 control), ATP/$MgCl_2$ (ATP control) and the deconvoluted compounds at 30µM. Representative curves obtained from two separate fluorescence profiling experiments are shown. **C)** Correlation of shift in HER3 Tm value induced by compounds tested at 4 concentrations (1, 3, 10 and 30µM; n=2). ApoHER3 was used for all DSF analyses and ATP/$MgCl_2$ was used as positive control. Proof-of-concept

compound AC3573 is highlighted. **D)** Correlation of shift in HER2 Tm value induced by compounds at 1, 3, 10 and 30μM for HER2 counter screening (n=2). ApoHER2 (HER2 in the absence of any ligand) was used for all DSF analyses and 1μM lapatinib was used as a positive control. Proof-of-concept compound AC3573 is highlighted.

Supplementary Figure 3

Dose-response effects of the 5 compounds identified in the single dose cell-based screen on NRG-induced HER3 phosphorylation and downstream signalling.

SK-BR-3 cells were serum-starved for 16 h and treated for 1 h with either DMSO, or the indicated concentrations of compounds A3 or A6 (**A**), or B1 or C5 (**B**) or G3 (**C**) or 1μM lapatinib and subjected or not to 15 min NRG stimulation (10nM). After lysis, whole-cell extracts were immunoblotted with the indicated primary antibodies for assessment of effects of compounds on NRG-induced signalling. Western blots shown are representative of two independent experiments. Compounds A3, A6, B1 and C5 prevented HER2-HER3 activation in response to NRG stimulation below 10μM, while compound G3 did not show any inhibition on HER3 downstream signalling below 30μM.

Supplementary Figure 4

In vitro kinase assay of the HER2 kinase domain against AC3573 performed at the Km for ATP conducted by ThermoFisher.

Supplementary Figure 5

HER3 peptide mapping for HDX-MS.

Peptide mapping was obtained by using non-deuterated samples in triplicate and only unique peptides present in all three data files were selected for deuterium uptake data analysis. Protein digests provided a list of 2,000 peptides. 146 peptides were selected for HDX analysis providing >96% sequence coverage with many overlapping peptides.

Supplementary Figure 6

AC3573 compound disrupts HER2-HER3 heterodimers but does not induce HER3 homodimers.

A) Differences in probability of HER2-HER3 nearest neighbour distances. Cluster measurements from STORM data taken from SK-BR-3 cells labelled with HER2-Alexa488

Affibody and HER3-CF640R SE Affibody (HER2-HER3) or NRG-CF640R SE (HER2-NRG) ± 1μM lapatinib or 30μM AC3573 compound. Graphs show near neighbour distribution of HER2 and HER3 molecules as Y condition (right-hand side) – X condition (top). A positive difference indicates that it is more likely to find a HER2 at the corresponding distance from a HER3 under Y condition than under X condition. **B)** HER3 cluster size (radii, left box plot) and number of HER3 molecules per cluster (right box plot) measurements from STORM data taken from SK-BR-3 cells labelled with HER3-CF640R SE Affibody in presence of 30μM AC 3573 compound or DMSO. The graphs show radii and number of molecules per cluster of 1007 clusters for DMSO treated cells and 630 clusters for AC3573 treated cells. The median values are shown. AC3573 compound did not alter either the size of HER3 clusters (median radii 29.3 nm for DMSO treated cells versus 29.7 nm for AC3573 treated cells), or the number of HER3 molecules per cluster which remained the same between control (DMSO) and AC3573-treated cells.

Supplementary Movie 1

A 360° rotation of HER3 bound to AMP-PNP (PDB:3KEX) showing the binding site of AC3573 (highlighted).

Supplementary Table 1

Detailed assay workflow for the identification of HER3 binders through the screening of a 107, 008 compound library by differential scanning fluorimetry.

Overview of the complete compound screening strategy, including all the compound screening stages and steps, experimental design, hit selection criteria, Tm of controls (ApoHER3 with no ligand and 200μM ATP/10mM $MgCl_2$ for HER3 screening, ApoHER2 without any ligand and 1μM lapatinib for HER2, as means ± SD calculated from all the plates assayed at each step of screening) and RZ factor (calculated from all the plates assayed at each step of screening).

Supplementary Table 2

Thermal shift results for the proof-of-concept compound AC3573 at the different stages of the screening cascade.

ΔTm values are reported as mean ± SD obtained from at least two independent experiments, except for the HER3 primary screen (N=1).

Supplementary Table 3

IC_{50} values of proof-of-concept compound AC3573 and compounds A3, A6, B1 and C5 against HER2 kinase domain were determined by an *in vitro* kinase assay performed at the Km for ATP (assay conducted by ThermoFisher).

Supplementary Table 4

Profiling of AC3573 at 1μM against a panel of 400 kinases performed at the ATP Km conducted by ThermoFisher.
